# Short-Term Test for Toxicogenomic Analysis of Ecotoxic Modes of Action in *Lemna minor*

**DOI:** 10.1021/acs.est.2c01777

**Published:** 2022-08-04

**Authors:** Alexandra Loll, Hannes Reinwald, Steve U. Ayobahan, Bernd Göckener, Gabriela Salinas, Christoph Schäfers, Karsten Schlich, Gerd Hamscher, Sebastian Eilebrecht

**Affiliations:** † Fraunhofer Attract Eco’n’OMICs, Fraunhofer Institute for Molecular Biology and Applied Ecology, Auf dem Aberg 1, Schmallenberg 57392, Germany; ‡ Institute of Food Chemistry and Food Biotechnology, Justus Liebig University Giessen, Heinrich-Buff-Ring 17, Giessen 35392, Germany; § Department Evolutionary Ecology and Environmental Toxicology, Goethe University Frankfurt, Max-von-Laue-Straße 9, Frankfurt am Main 60438, Germany; ∥ Department of Food and Feed Safety, Fraunhofer Institute for Molecular Biology and Applied Ecology, Schmallenberg 57392, Germany; ⊥ NGS-Services for Integrative Genomics, University of Göttingen, Göttingen 37077, Germany; # Department Ecotoxicology, Fraunhofer Institute for Molecular Biology and Applied Ecology, Schmallenberg 57392, Germany

**Keywords:** transcriptomics, proteomics, biomarkers, functional annotation, HMG-CoA reductase inhibition, PSII inhibition, Lemna minor

## Abstract

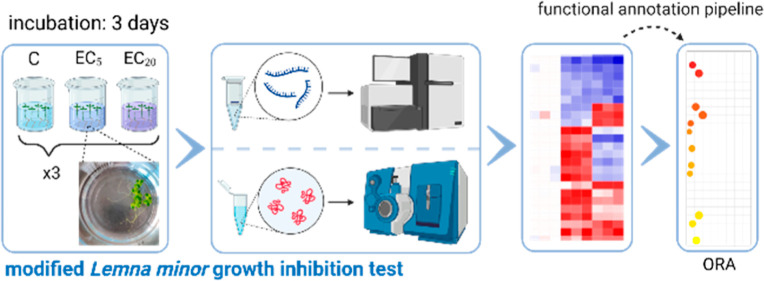

In the environmental risk assessment of substances, toxicity to aquatic plants is evaluated using, among other methods, the 7 day*Lemna* sp. growth inhibition test following the OECD TG 221. So far, the test is not applicable for short-term screening of toxicity, nor does it allow evaluation of toxic modes of action (MoA). The latter is also complicated by the lack of knowledge of gene functions in the test species. Using ecotoxicogenomics, we developed a time-shortened 3 day assay in*Lemna minor* which allows discrimination of ecotoxic MoA. By examining the changes in gene expression induced by low effect concentrations of the pharmaceutical atorvastatin and the herbicide bentazon at the transcriptome and proteome levels, we were able to identify candidate biomarkers for the respective MoA. We developed a homology-based functional annotation pipeline for the reference genome of*L. minor*, which allowed overrepresentation analysis of the gene ontologies affected by both test compounds. Genes affected by atorvastatin mainly influenced lipid synthesis and metabolism, whereas the bentazon-responsive genes were mainly involved in light response. Our approach is therefore less time-consuming but sensitive and allows assessment of MoA in *L. minor*. Using this shortened assay, investigation of expression changes of the identified candidate biomarkers may allow the development of MoA-specific screening approaches in the future.

## Abstract 2

By combining a shortened growth inhibition test with transcriptomics and proteomics, a method for rapid assessment and differentiation of modes of action in *L. minor* was developed.

## Introduction

For the registration of a chemical under REACH (Registration, Evaluation, Authorisation and Restriction of Chemicals) or of an active ingredient in pesticides, pharmaceuticals, or biocides, the testing for effects on aquatic organisms is a registration requirement. Due to the increasing number of these anthropogenic substances on the market, the development of rapid and meaningful test systems to assess the potential hazard in the environment is becoming more and more important.^1^ Standardized ecotoxicity tests for the environmental hazard assessment of xenobiotics have already been established for a variety of aquatic model organisms, including *Lemna minor.* However they generally do not allow rapid screening or discrimination of harmful modes of action (MoA). The growth inhibition test in*L. minor*according to the Organization for Economic Cooperation and Development (OECD) Test Guideline (TG) no. 221, for example, captures changes in plant growth as an endpoint and takes 7 days.^2^

Our study aimed to develop an abbreviated test that would allow an identification of gene expression biomarkers for discrimination of MoA in*L. minor*beyond the classical endpoints. To this end, we developed an abbreviated version of the OECD TG 221 and combined it with the detection of compound-induced gene expression changes at the transcriptome and proteome levels. OMICs, as a non-target method, offers the possibility of comprehensive detection of molecular fingerprints and informative biomarker candidates.^3,4^ Although a first draft genome for this species was published back in 2015, transcriptome methods have rarely been applied to*L. minor*.^5^ For example, Wang et al. investigated molecular responses of*L. minor* to ammonia (NH_4_^+^),^6^ and Li et al. recorded transcriptome changes after exposure to the EC_50_ of the pesticide imazamox.^7^ Both studies used the 7 day OECD guideline test for this purpose. Proteomic studies on*L. minor*have been largely lacking. Moreover, due to the previously non-functionally annotated reference genome, it has been difficult to read out functional information from OMICs results, which may provide information on MoA, for example. Our approach here aimed to detect predictive biomarker candidates for mechanisms of action at an early stage using OMICs, which can then be screened in the future using rapid analytical methods, such as reverse transcription-quantitative polymerase chain reaction (RT-qPCR) or fluorescence-based methods.

We used the drug atorvastatin and the herbicide bentazon as reference substances to establish our abbreviated, MoA-specific assay approach. Atorvastatin is one of the most commonly used statins in human medicine,^8^ which inhibits 3-hydroxy-3-methyl-glutaryl-coenzyme A reductase (HMGR), the key enzyme in human cholesterol synthesis, and thus has cholesterol- and lipid-lowering effects.^9^ Plants also possess an HMGR very similar to humans, which is involved in phytosterol synthesis through the mevalonic acid (MVA) pathway.^10,11^ A phytotoxic effect of atorvastatin has already been demonstrated in *Lemna gibba*, making it a promising candidate for our studies.^12^ Bentazon is a herbicide that inhibits photosystem II (PSII) and thus photosynthesis in plants.^13^ In addition, bentazon has also been associated with inhibition of HMGR in a previous study.^14^

To detect early changes in gene expression induced by these two reference compounds, a shortened assay for growth inhibition of *L. minor* was developed, which integrates systems biology methods. For this purpose, the gene expression profiles after shortened exposure to low effect concentrations (ECs) of both substances was recorded, compared, and functionally evaluated. A robust and comprehensible pipeline was established and applied for functional annotation of the *L. minor* reference genome. The assay approach was evaluated based on the identifiability of candidate biomarkers and on the distinguishability of the MoA of both reference compounds.

## Materials and Methods

### Test Substances

The HMG-CoA reductase inhibitor atorvastatin calcium (CAS 134523-03-8, purity >95%) and the photosynthesis inhibitor bentazon (CAS 25057-89-0, purity ≥98%) were purchased from abcr and Sigma-Aldrich, respectively. All test solutions and dilutions used were prepared with axenic Steinberg medium, made from a 10-fold-concentrated stock solution according to OECD TG 221^2^ the day before test start at pH 5.5 ± 0.2. Solubilization was done by stirring for 2 h (atorvastatin) or 1 h (bentazon) followed by 15 min in an ultrasonic bath. Preparation of atorvastatin solutions was carried out taking into account that the substance is a calcium salt. All concentrations and effects therefore refer to the active substance. On the day of the test start, all test solutions were prepared as a dilution series of the highest test concentration in Steinberg medium.

### *L. minor* Culture and Determination of ECs

**Figure 1:**
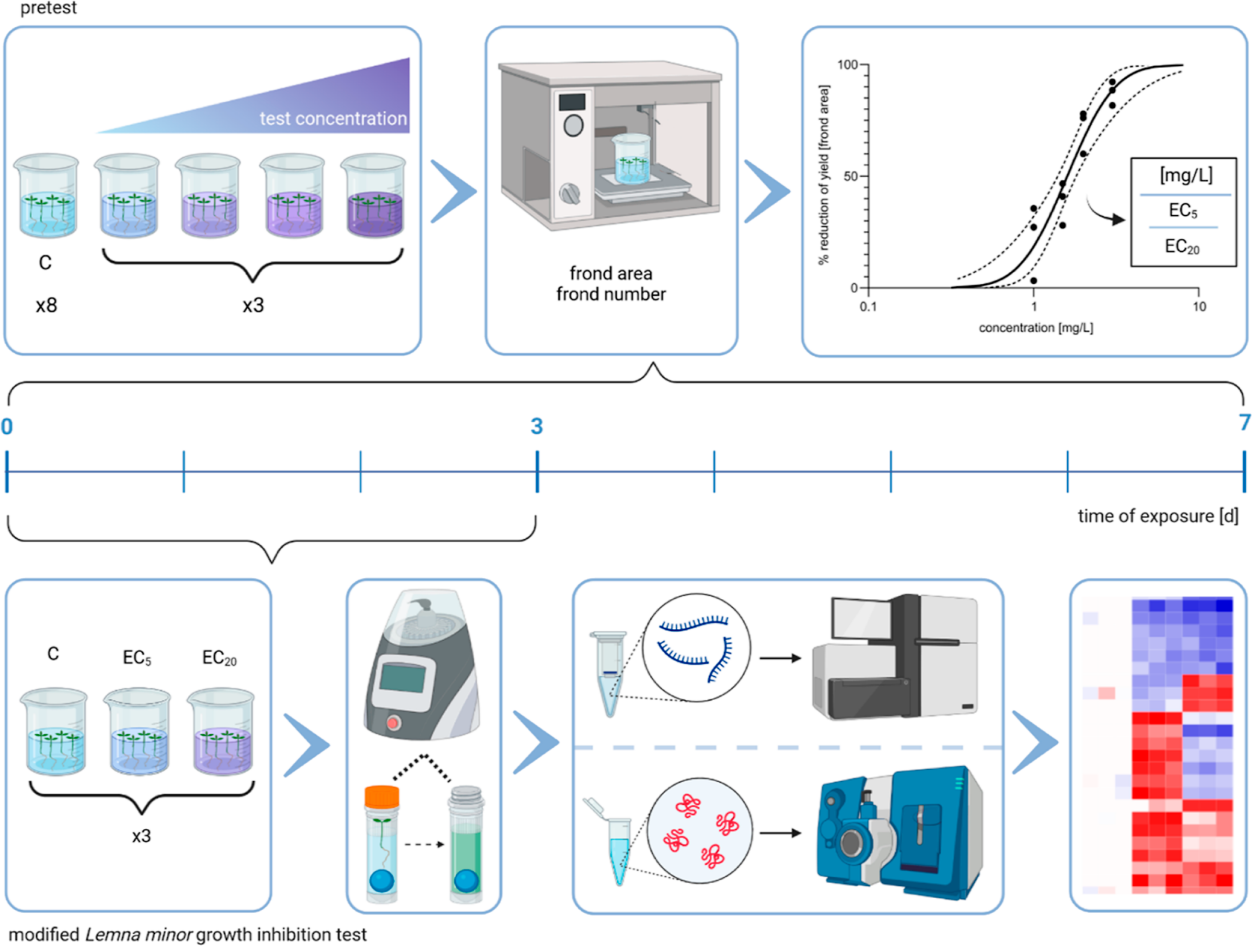
Schematic view and timelines of the pretest (top) and the modified *L. minor* growth inhibition test (bottom) workflows. The pretest followed the instructions of OECD TG 221 and was conducted with four test concentrations (three replicates) and a control condition (eight replicates) over a period of 7 days. After measuring the frond area and frond number, concentration–response curves were generated. Subsequently, the EC_5_ and EC_20_ were used as test conditions for the modified *L. minor* growth inhibition test, which was shortened to 3 days. At day 3, the plant material obtained was used for RNA and protein extraction for transcriptome and proteome analysis. Created with BioRender.com.

In order to identify suitable test concentrations for the modified *L. minor* growth inhibition test, ECs were determined for both test substances in pretests following OECD TG 221.^2^ Briefly, *L. minor* were exposed to four successive concentrations of each test substance and an appropriate water control for 7 days under static conditions. Pretest concentrations for each substance were chosen based on the available literature.^12,15,16^ Each test was performed with three replicates, while the control was performed with eight replicates using a total volume of 150 mL per test vessel (Figure 1). Four healthy plants with three fronds each from a pre-culture of at least 1 week were used for each replicate. At the beginning and at the end of the pre- and modified inhibition test, the pH was measured for all samples (Tables S1 and S2). Plants were exposed under continuous light (85–135 μE m^–2^ s^–1^) at 24 ± 2 °C in a random arrangement using a Multitron Pro growth chamber (Infors HT) (Table S2).

At the beginning of the test and after 7 days and at two time points in between, the area and number of the fronds were measured using a *Count & Classify v6.8* image analysis system (medeaLAB, Erlangen, Germany). Concentration–response curves were constructed by plotting the percent reduction in yield for both parameters after 7 days of exposure against the concentration of the test substance. Data analysis and calculation of ECs were performed by probit analysis using a linear maximum likelihood regression model (ToxRat v.3.0.0 software; ToxRat Solutions GmbH, Alsdorf, Germany).

### Modified Short-Term *L. minor* Growth Inhibition Test

For the early identification of substance-induced gene expression changes, a shortened version of the OECD TG 221^2^ was performed with *L. minor* exposed to the EC_5_ and the EC_20_ of each test substance identified in the pretests. The shortened exposure lasted 72 h under static conditions, and the test was conducted in three replicates per exposure condition and control (Figure 1). The incubation conditions were identical to those of the guideline and were already described above. At the end of the test, the overgrown fronds were separated, and the number and area of the fronds were determined as previously described, before total RNA and protein were extracted for subsequent transcriptome and proteome analysis.

### Chemical Analysis

The concentrations of atorvastatin and bentazon in Steinberg medium were determined by chemical analysis. Briefly, the aqueous samples were amended with methanol and further diluted if necessary. The samples were then directly analyzed by ultra-high-performance liquid chromatography coupled with tandem mass spectrometry (UHPLC-MS/MS).

### RNA Extraction

For each sample, total RNA was isolated and purified from 25 mg of the plant material following an optimized version of the manufacturer’s protocol of the *RapidPURE RNA Plant Kit* (MP Biomedicals, Illkirch, France). A detailed description is given in the Supporting Information. The purity and concentration of the RNA were assessed using a Nanodrop 2000 instrument (Thermo Scientific), and RNA integrity was determined using a Bioanalyzer 2100 (Agilent Technologies). To assure high RNA quality, only samples with RNA Integrity Numbers (RINs) > 7.0 and a purity of A_260/230_ > 1.6 and A_260/280_ > 2.0 were selected for sequencing.

### Transcriptomics

Poly(A)+ RNA was purified, fragmented, and transcribed into cDNA for library preparation using the *TruSeq RNA Library Prep Kit (v2)* (Illumina, UK) following the manufacturer’s instructions. Sample libraries were sequenced on an Illumina HiSeq 4000 system in the 50 bp single read mode with approximately 30 million raw reads per sample. In short, after adapter-trimmed sequence quality and contamination checks via FastQC (v0.11.5)^17^ and FastQ Screen (v0.14.1),^18^ reads were mapped to *L. minor* reference genome 2019v2^5^ (www.lemna.org) using STAR (v2.7.8a)^19^ and the respective genome annotation file.^20^ A MultiQC^21^ sequence read and alignment quality report is provided in the Supporting Information.

Reads were counted with featureCounts (v2.0.1).^22^ Raw sequencing files and processed gene files were deposited in the ArrayExpress database under accession numbers E-MTAB-11459 (atorvastatin) and E-MTAB-11460 (bentazon).^23^

Gene count library normalization and differential gene expression analysis (DGEA) were conducted in R^24^ using DESeq2 (v1.30.0).^25^ Low abundant counts were removed prior to DESeq2’s median of ratios normalization and statistical testing for significant expression differences using Wald’s *t*-test and Benjamini–Hochberg (BH) correction with IHW for multiple testing.^26^ To improve the effect size signal-over-noise ratio, obtained log_2_-fold change (lfc) values were shrunk using the *apeglm* method.^27^ For comparison of each treatment against the respective control group, an effect size cutoff (LFcut) was determined as the top 25% quantile of absolute non-shrunk lfc values *LFcut = quantile[abs(lfc), 0.75]*. A gene was considered differentially expressed (DEG) when (a) statistical (padj ≤ 0.05) and (b) effect size cut-off criteria {*abs[apeglm(lfc)] ≥ LFcut*} were met, as described previously.^28^

### Protein Extraction, Digestion, and Peptide Labeling

Total protein was extracted simultaneously with RNA using the *RapidPURE RNA Plant Kit* (MP Biomedicals, Illkirch, France). For this, the protein-containing flow-through from RNA extraction was subjected to acetone precipitation. Precipitated proteins were resolubilized in 50 mM *triethylammonium bicarbonate* (TEAB) containing 4% 3-[(3-cholamidopropyl)dimethylammonio]-1-propanesulfonate, 2 M thiourea, and 6 M urea at pH 8.2, before buffer was exchanged to 100 mM TEAB containing 0.2% sodium dodecyl sulfate and 2 M urea at pH 8.4 via 30 kDa molecular weight cut-off filters (Merck Darmstadt, Germany) for quantification, using the *Pierce BCA Protein Assay Kit* (Thermo Scientific, USA). The subsequent workflow for labeling tryptic-digested protein samples TMT-6plex (Thermo Scientific, USA) followed the manufacturer’s recommendations.

### Proteomics

For quantitative proteomics, 500 ng combined TMT-labeled peptides were injected replicate wise onto a nanoACQUITY UPLC C18 Trap Column, before being separated on a nanoACQUITY reversed-phase analytical column (Waters, Massachusetts, USA) using a linear gradient from 3 to 97% (v/v) of 90% (v/v) acetonitrile in 0.1% (v/v) formic acid for 170 min with a flow rate of 300 nL/min. Eluted peptides were analyzed on a Thermo Fisher Q Exactive mass spectrometer (Thermo Fisher, Waltham, USA) as described previously.^29,30^

The resulting MS/MS data were processed using the MaxQuant search engine (v.2.0.1.0).^31^ Tandem mass spectra were matched to a custom protein database with the predicted protein sequence from the *L. minor* reference genome combined with duckweed-related protein sequences (pro- and eukaryotic origin) obtained from UniProt (search term “duckweed”). Furthermore, a common laboratory contaminant protein list was provided for the PSM search. DEG proteins were identified using the MSstatsTMT R package (v.2.2.0)^32^ on the basis of three technical replicate measurements of three biological replicates per condition. Statistical significance was assessed by comparing treatment to the non-treated control using the MSstatsTMT’s implemented linear mixed model with a moderated *t*-statistic. Proteins were considered statistically significantly regulated for BH-corrected *p*-values (padj) < 0.05^33^ with degrees of freedom ≥ 6. The mass spectrometry proteomics data have been deposited in the ProteomeXchange Consortium via the PRIDE partner repository^34^ with the data set identifiers PXD031680 (atorvastatin) and PXD031679 (bentazon).

### Functional Genome Annotation and Overrepresentation Analysis

A complementary approach using the basic local alignment search tool (BLAST)^35^ and evolutionary genealogy of genes: non-supervised orthologous groups (eggNOG)^36^ was applied to annotate the *L. minor* reference genome (2019v2) with gene ontology (GO) terms and gene descriptors based on protein sequence homology. A detailed workflow description is given in the Supporting Information, and a general overview is shown in Figure 5. Briefly, a multi-fasta file listing the coding sequence per gene was extracted from the reference genome via the respective GTF file [Zenodo 6045874] and translated into amino acid sequences. For the BLAST-based annotation, a local search database was created from the UniProt database^37^ containing peptide sequence information from closely related plant species and duckweed-associated microorganisms. Translated *L. minor* sequences were subjected to a BLASTP search against this local database, and each gene was annotated with the UniProt ID of the best hit scored by % alignment for each reference species. Results were cleaned for non-plant-related top hits and alignment lengths < 20 amino acids and alignment similarities < 35%. Each *L. minor* gene ID of these cleaned results was then annotated with the combined set of unique GO terms associated with the matched plant-related UniProt IDs. Additionally, translated *L. minor* sequences were subjected to the eggNOG annotation with default settings.^38^ All plant-related matches were then combined in a single data frame with corresponding GO terms from which the org.Lminor.eg.db annotation package was constructed. Based on this custom-built annotation package, overrepresentation analysis (ORA) was performed in R using clusterProfiler v3.18^39^ as described in the Supporting Information.

## Results and Discussion

### Identification of ECs of Atorvastatin and Bentazon in *L. minor*

To determine low ECs for ecotoxicogenomic assessment in a modified ecotoxicity test with *L. minor*, preliminary range-finding tests were performed. The use of low ECs for ecotoxicogenomic analyses aimed to capture molecular effects of the respective MoA and to exclude systemic effects, which may occur at higher ECs, as far as possible. For each test substance, the effect of four concentrations was observed based on OECD TG 221, which were based on a literature review.^12,15,16^

**Figure 2:**
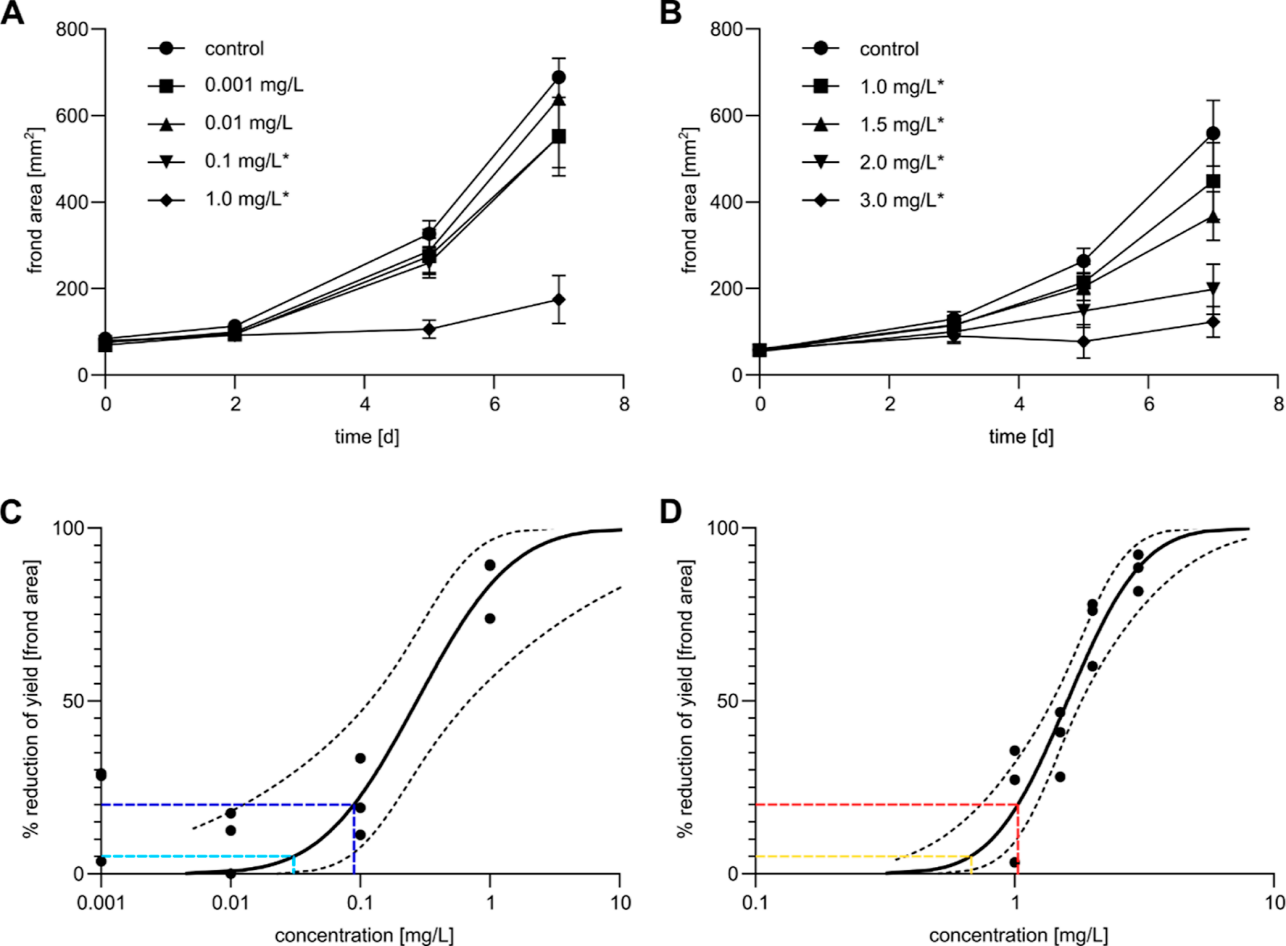
Pretest for detecting ECs of atorvastatin and bentazon according to OECD TG 221. (A,B) Time-dependent course of the frond area at different exposure concentrations of atorvastatin (A) and bentazon (B). Statistically significant changes at day 7 compared to the control are indicated by an asterisk (Williams Multiple Sequential *t*-test). The standard deviation is given as an error bar. (C,D) Concentration–response curve of frond area yields reduction after exposure to atorvastatin (C) and bentazon (D) on day 7. The EC_5_ is colored in light blue and yellow, and the EC_20_ is colored in dark blue and red.

Among the analyzed endpoints, frond area was found to be the most sensitive parameter (Tables S3 and S4), which was therefore used to determine ECs for further ecotoxicogenomic test development. For atorvastatin (Figure 2A), the two highest, and for bentazon (Figure 2B), all of the test concentrations resulted in statistically significant changes in frond area, as determined by the Williams Multiple Sequential *t*-test. Concentration–response curves were generated (Figure 2C,D), which were then used to calculate ECs (Table S3). Whereas in the case of atorvastatin, ECs were distributed over more than two orders of magnitude until a maximal effect was achieved (Figure 2C), in the case of bentazon, the maximal effect was achieved much more rapidly over a concentration range of less than one order of magnitude (Figure 2D). In contrast, atorvastatin ECs (Figure 2C and Table S3) were by far lower than those of bentazon both in terms of mass concentration and molarity (Figure 2D and Table S3), indicating a significantly higher toxic potency of the pharmaceutical as compared to the herbicide.

Until now, only one previous study has investigated the toxicity of atorvastatin in *L. minor*.^40^ Klementová et al. found no significant effects up to a concentration of 200 mg/L, which is in contrast to the results of our study. The authors of this study worked with aqueous solutions of atorvastatin and not, as we did, with its calcium salt. The solubility limit of atorvastatin in water is 1 μg/L and was clearly exceeded in all nominal concentrations tested by Klementová et al., suggesting that the actual concentrations may have been much lower. In addition, Klementová et al. described significant effects of photoproducts of atorvastatin in their study. Under exposure conditions according to OECD guideline test 221, plants are permanently illuminated, so our study did not distinguish between the effects of the parent compound and those of possible bioactive photoproducts. Therefore, our results do not necessarily contradict those of Klementová et al. Furthermore, our data in *L. minor* agree well with the phytotoxicity observed in the closely related species *L. gibba* in a previous study, which observed an EC_50_ value of 0.24 mg/L for the frond number endpoint (Table S4).^41^

For bentazon, several previous studies investigated toxicity in *L. minor*, predominantly according to the ISO 20079 standard, which has largely identical requirements to those of the OECD TG 221.^2,42^ Munkegaard et al. observed an EC_50_ of 2.94 mg/L on assessing the relative growth rate of frond area.^15^ Similarly, Cedergreen and Streibig. identified an EC_50_ of 2.56 mg/L.^16^ These previously identified ECs were in the same order of magnitude as the EC_50_ observed in our study (Table S3), validating our experimental setting.

### Gene Expression Signatures of Atorvastatin and Bentazon in *L. minor*

Since our study aimed to discriminate ecotoxic MoA based on gene expression profiles in a shortened *L. minor* growth inhibition assay, EC_5_ and EC_20_ obtained with the regular OECD TG 221 were chosen as low ECs to exclude systemic effects as much as possible. Thus, the shortened test was conducted with nominal concentrations of 0.03 (EC_5_) and 0.09 mg/L (EC_20_) for atorvastatin and 0.7 (EC_5_) and 1.0 mg/L (EC_20_) for bentazon. Chemical analysis employing UHPLC-MS/MS yielded recoveries between 90 and 103% of nominal test concentrations, so nominal concentrations are referenced below (Table 1). After a treatment period of 3 days, gene expression changes were investigated by transcriptomics and proteomics.

**Table 1:** Nominal and Measured Concentrations of Control and Test Solutions for Both Substances

	atorvastatin			bentazon		
[μg a.s./L]	nominal	measured	recovery	nominal	measured	recovery
control	0			0		
EC_5_	30.0	27.1	90.3%	700.0	716.5	102.4%
EC_20_	90.0	84.6	94.0%	1000.0	922.8	92.3%

**Figure 3:**
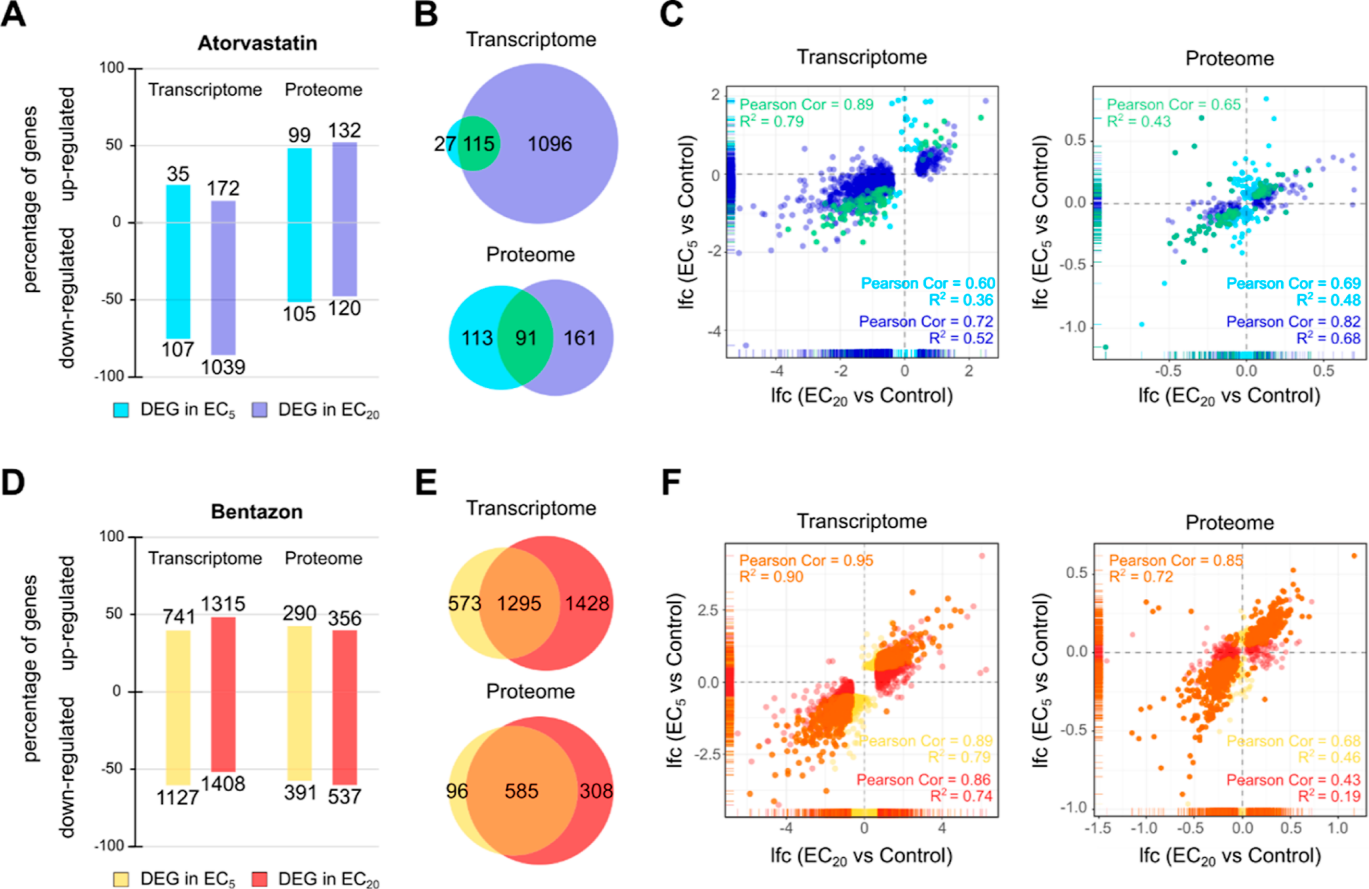
Gene expression changes in *L. minor* induced by exposure to EC_5_ and EC_20_ of atorvastatin and bentazon after 3 days. (A) Percentages of the up- and downregulation of DEGs at the transcriptome and proteome level after exposure to EC_5_ (light blue) and EC_20_ (dark blue) of atorvastatin compared to the control. The number of up- and downregulated genes is indicated as bar labels. (B) Venn diagrams showing the numbers of DEGs after exposure to EC_5_ (light blue) and EC_20_ (dark blue) of atorvastatin and their intersection (green) at the transcriptome (top) and the proteome level (bottom). (C) Scatter plots showing the correlation of differential gene expression between exposure to the EC_5_ and EC_20_ of atorvastatin at the transcriptome (left) and the proteome level (right) comparing their lfc values. Coloring as in (B). (D) Percentages of the up- and downregulation of DEGs at the transcriptome and proteome level after exposure to EC_5_ (yellow) and EC_20_ (red) of bentazon compared to the control. The number of up- and downregulated genes is indicated as bar labels. (E) Venn diagrams showing the numbers of DEGs after exposure to EC_5_ (yellow) and EC_20_ (red) of bentazon and their intersection (orange) at the transcriptome (top) and the proteome level (bottom). (F) Scatter plots showing the correlation of differential gene expression between exposure to the EC_5_ and EC_20_ of bentazon at the transcriptome (left) and the proteome level (right) comparing their lfc values. Coloring as in (E).

For both test compounds, we observed a concentration-dependent behavior of the gene expression, both in terms of the number of DEGs and the strength of their regulation (Figure 3), with more DEGs and generally higher lfc values in response to EC_20_ compared to EC_5_.

In the case of atorvastatin, 142 DEGs were detected at the transcriptome level by EC_5_ and 1211 by EC_20_, of which the majority (75 and 86%, respectively) were downregulated (Figure 3A). At the proteome level, 204 and 252 DEGs were identified in response to EC_5_ and EC_20_, respectively, of which half (51 and 48%, respectively) were downregulated. At the transcriptome level, 81% (115 genes) of DEGs responsive to EC_5_ were also DEG after exposure to EC_20_ (Figure 3B). At the proteome level, predominantly those proteins were detected that were most highly expressed at the RNA level (Figure S7A). Here, the intersection between EC_5_ and EC_20_ consisted of 45% (91 genes) of the DEGs responding to EC_5_. Remarkably, such genes that were detected at the transcriptome and proteome levels and were strongly differentially regulated at the RNA level were also significantly regulated in the same direction at the protein level (Figure S7B). The common DEG sets of EC_5_ and EC_20_ treatment conditions, representing early and consistently regulated genes, were defined as core DEG sets for each test compound. The lfc values induced by EC_5_ and EC_20_ exposure showed a strong and moderate positive correlation for the atorvastatin core DEG sets at the transcriptome and proteome levels, respectively [Pearson correlation = 0.89 (transcriptome) and 0.65 (proteome)] (Figure 3C), making them a source of early biomarker candidates for inhibition of HMG-CoA reductase in*L. minor*.

In the case of bentazon, 1868 and 2723 DEGs were identified at the transcriptome level after exposure to EC_5_ and EC_20_, respectively, about half of which (60 and 52%, respectively) were downregulated (Figure 3D). Also, in the case of bentazon, the gene products that were most highly expressed at the RNA level were detected at the proteome level (Figure S7C). Here, 681 and 893 DEGs were induced by EC_5_ and EC_20_, respectively, showing a comparable direction of regulation as in the transcriptome analyses (57 and 60% downregulation, respectively). A proportion of 69% (1295 genes) and 86% (585 genes) of the DEGs responding to EC_5_ at the transcriptome and proteome levels, respectively, also responded to EC_20_ (Figure 3E). As with atorvastatin, genes detected together in the transcriptome and proteome that were most highly regulated at the RNA level were also significantly regulated in the same direction at the protein level (Figure S7D). These core DEG sets of bentazon also showed strong positive correlations when comparing gene expression changes induced by EC_5_ and EC_20_ [Pearson correlation = 0.95 (transcriptome) and 0.85 (proteome)] and therefore contain early biomarker candidates for photosynthesis inhibition (Figure 3F).

Although few previous studies have analyzed transcriptomic changes induced by various stressors in *L. minor*,^5−7^ proteomic data are notably lacking for molecular analysis in this species. Accordingly, transcriptome and proteome data have never been integrated in this test organism. Validation of trends in gene expression at the other level would strengthen the biological relevance of the results and facilitate biomarker identification. For such validation, we compared the expression changes of the DEGs after EC_5_ and EC_20_ exposure and the core DEGs of both compounds at the proteome level with those at the transcriptome level (Table S5 and Figure S8). The positive quadrant count ratios of these comparisons ranged from 0.37 to 1.00, clearly indicating that the vast majority of DEGs were regulated in the same direction at both levels. Thus, our data clearly demonstrate that both OMICs methods are applicable to a shortened growth inhibition assay in *L. minor* to generate comprehensive core DEG sets affected by low ECs as toxicogenomic fingerprints.

**Figure 4:**
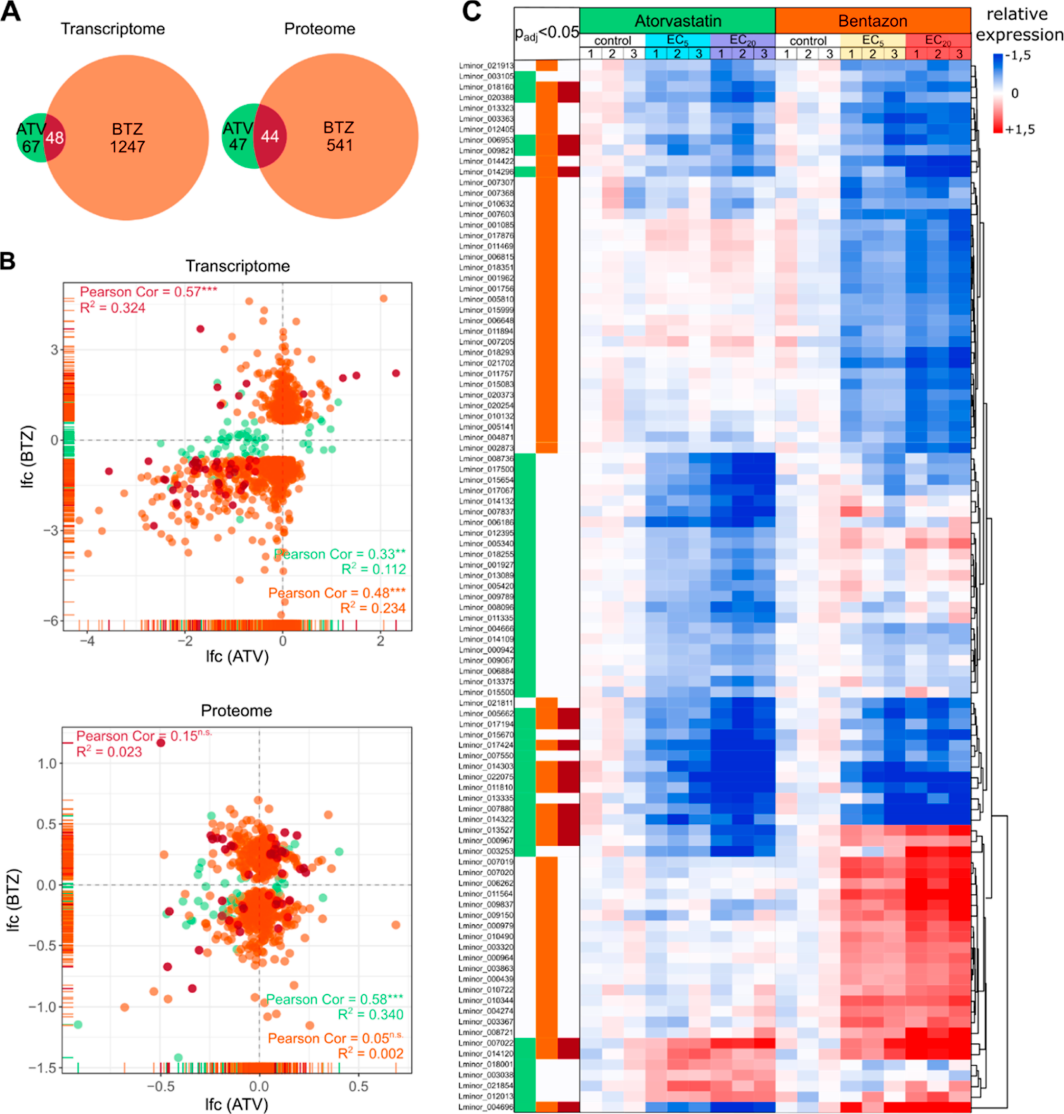
Comparison of gene expression signatures induced by atorvastatin and bentazon in *L. minor*. (A) Venn diagrams showing the numbers of DEGs in the intersections of EC_5_ and EC_20_ exposures after atorvastatin (ATV, green) and bentazon (BTZ, orange) exposure at the transcriptome (left) and the proteome level (right). The intersections are colored in dark red. (B) Scatter plots comparing the lfc values of DEGs in the intersections of EC_5_ and EC_20_ exposures after atorvastatin and bentazon exposure at the level of the transcriptome (top) and proteome (bottom). Coloring as in (A). *p < 0.05, **p < 0.01, ***p < 0.001, and n.s. “not statistically significant”. (C) Heatmap showing the relative expression of the top 50 DEGs in the intersections of EC_5_ and EC_20_ exposures for both substances at the transcriptome level, based on their mean expression under the control condition. Red color indicates upregulation and blue downregulation of a gene as compared to the control. The color code on the top of each column illustrates the test condition. Columns indicate biological replicates (1–3) per condition. Genes were clustered by Euclidean distance. The color code on the left assigns the genes to the DEG sets defined in (A).

To assess whether these fingerprints of each compound could serve as a basis for MoA discrimination, we next compared the identified core DEG sets of atorvastatin and bentazon. While atorvastatin acts as an HMGR inhibitor in humans and plants,^12^ the herbicide bentazon interferes with photosynthesis by inhibiting plant PSII.^43^ However, previous studies also suggested that bentazon has inhibitory properties related to HMGR activity.^14^ In view of these potentially partially concordant MoA of both test substances, it was particularly interesting to identify similarities and differences in their gene expression profiles. At the transcriptome level, the core DEG sets of atorvastatin and bentazon overlapped in a total of 48 genes, which accounted for 42% of the atorvastatin signature and 4% of the bentazon signature (Figure 4A). At the level of the proteome, the intersection of the core DEG sets totaled 44 genes, which corresponded to a 48% share of the atorvastatin signature and an 8% share of the bentazon signature, while in the case of the transcriptome, the genes that were jointly targeted by both compounds showed a positive correlation when comparing the two compounds (Pearson correlation = 0.57, *p* ≤ 0.0001), and the genes of the intersection were not significantly correlated at the proteome level (Pearson correlation = 0.15, *p* = 0.3229) (Figure 4B). Remarkably, the signatures of both substances that were not part of the intersection behaved in a substance-specific manner, that is, their expression was predominantly not regulated by the respective other substance. Therefore, the common subset of core DEGs of both compounds at the transcriptome level may result from and indicate partial concordance in MoA, such as partial HMGR inhibition whereas those of the intersection of core DEGs at the proteome level and compound-specific core DEG sets may allow discrimination of both MoA.

To provide a focus on robustly expressed biomarker candidates for both MoA, we extracted the 50 topmost expressed core DEGs of both compounds in terms of their expression levels under the control condition (Figure 4C). The resulting signatures allow clear discrimination of the molecular effects of atorvastatin and bentazon, and the shared gene clusters represent a minor proportion of each signature. Nevertheless, the common signatures show similar regulation in the vast majority of genes. Of particular interest for the selection of potential discriminatory biomarkers are genes that are differentially regulated by the two compounds. Examples of such promising biomarker candidates include the genes Lminor_013527, Lminor_000967, and Lminor_004696.

### Functional Classification of Molecular Effects of Atorvastatin and Bentazon

**Figure 5:**
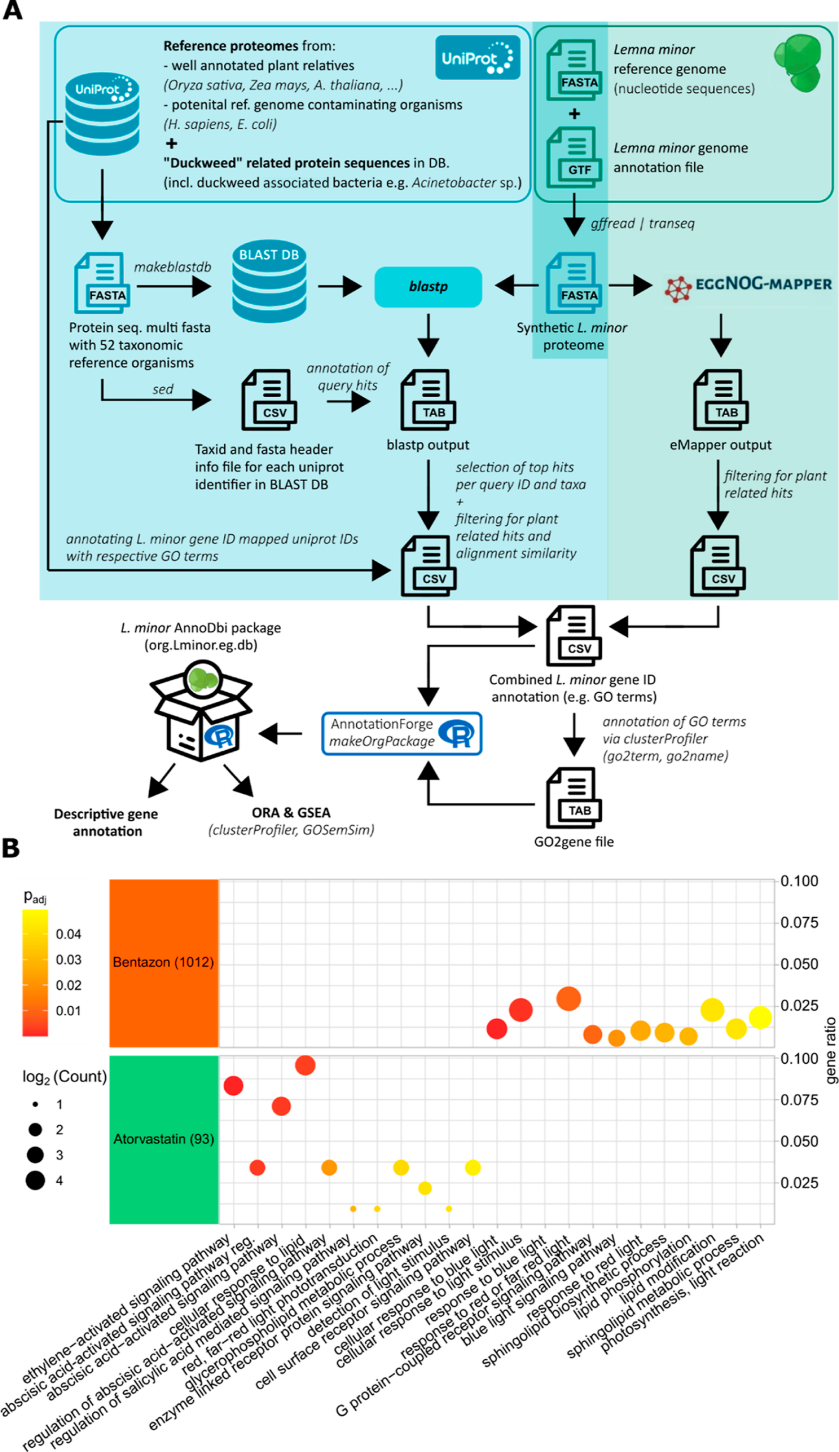
Functional analysis of gene expression responses induced by exposure to low ECs of atorvastatin and bentazon in*L. minor*. (A) Bioinformatic pipeline for functional gene annotation in *L. minor*. A complementary approach applying BLAST^35^ and eggNOG^36^ was used to assign genes of the*L. minor* reference genome to GO terms. Detailed information can be found in the Materials and Methods section and in the Supporting Information. (B) ORA of the DEGs in the intersections of EC_5_ and EC_20_ exposure to atorvastatin (left) and bentazon (right) using the GO biological function. The log_2_-converted gene count for each ontology term is indicated as bubble size, and the gene ratio (geneR) is plotted on the *x*-axis. *P*-values are given as a color code.

To gain insights into the functional processes affected by the two test compounds, we functionally annotated the *L. minor* reference genome to enable ORA of DEG sets with respect to gene ontologies such as biological processes, molecular functions, or cellular compounds. To this end, we developed a homology-based bioinformatics workflow that allowed GO terms of closely related plant proteins to be mapped to the corresponding*L. minor*gene IDs (Figure 5A). About 99.5% of*L. minor*genes were assigned to duckweed-related taxa or other plants through our pipeline, whereas only a small fraction of 0.5% had the highest homology with bacterial proteins, which might be due to symbiotic living prokaryotes (Figure S9). Nevertheless, this clear assignability of genes demonstrates the robustness of our annotation approach, which is an important prerequisite for generating meaningful ORA results. The resulting*L. minor*AnnoDbi package was used for ORA analysis of the identified core DEG sets of atorvastatin and bentazon.

When we focused on lipid- and light-related biological processes, we identified a number of significantly impaired metabolic pathways by one or both test substances (Figure 5B). While light-related processes were predominantly affected by bentazon, different lipid-related processes were affected by either test substance, consistent with the partial agreement in MoA mentioned above.

Biological processes affected by atorvastatin were predominantly related to lipid metabolic processes, reflecting HMGR-inhibitory MoA leading to lipid and sterol deficiency. In particular, the cellular response to lipids, but also several biological processes related to signaling pathways activated by abscisic acid (ABA), was significantly affected by atorvastatin exposure. These results are consistent with those of previous studies reporting ABA-mediated regulation of HMGR expression or activity.^44−46^ ABA is an isoprenoid hormone synthesized via the chloroplastic 2-*C*-methyl-*D*-erythritol 4-phosphate (MEP) pathway. In addition to the MEP pathway, the mevalonate (MVA) pathway is the major metabolic pathway for the biosynthesis of isoprene precursors. One of the key enzymes of the MVA pathway is HMGR. MEP and MVA pathways operate in parallel and compensate for each other through intermediates such as isopentyl diphosphate.^12^ Therefore, the observed changes in ABA-mediated signaling after atorvastatin exposure may represent a response to impaired synthesis of isoprene precursors due to HMGR inhibition by linking the MEP and MVA pathways. Such observations were previously made in*Arabidopsis thaliana*after lovastatin treatment, where the carotenoid and chlorophyll content (MEP products) was increased after HMGR inhibition.^47^ A possible link between carotenoid synthesis and ABA signaling in plants arises from the fact that the carotenoid zeaxanthin is a precursor of ABA. Among the biological processes most affected was the ethylene-activated signaling pathway. Ethylene treatment was shown to upregulate HMGR expression in*Dioscorea zingiberensis*, suggesting a role for this enzyme in ethylene signaling which is consistent with our results. In addition, atorvastatin also affected genes involved in a limited number of light-related processes such as light stimulus detection or red light phototransduction. A previous study by Zheng et al. found a light-induced reduction in HMGR activity in grapevine, suggesting a direct or indirect role of HMGR in light sensing, which may also be reflected in our observations.^48^

However, the response to light was impaired, especially by bentazon. The biological processes most strongly regulated by bentazon were cellular response to blue light and the light stimulus, response to red or far-red light, and the blue light signaling pathway. Bentazon is an inhibitor of PSII, which is the most light-sensitive component of the photosynthetic apparatus. Treatment of plants with PSII inhibitors thus causes them to die faster when exposed to light than in the dark.^49^ However, the toxic MoA of PSII herbicides is not directly triggered by light but by damage to cells due to an excessive amount of non-released light energy. Normally, absorbed energy is transferred from chlorophyll to PSII within the electron transport chain of photosynthesis.^50^ Since blocking PSII results in a break in this chain, the absorbed and short-lived energy must be dissipated by other means. Although excess energy is normally dissipated by carotenoids, in the case of PSII inhibition, the amount of energy is too high, resulting in the formation of lipid radicals that lead to lipid peroxidation of the membrane bilayer and ultimately to plant death.^49^ These processes are also reflected in our ORA results, where bentazon caused changes in various lipid-related biological processes such as sphingolipid metabolism, lipid modification, or lipid phosphorylation. These results are also supported by recent studies by Czékus et al. where bentazon treatment caused increased lipid peroxidation and ion leakage in soybean and common ragweed.^43^

Based on our gene expression profiles and the biological functions affected by the respective test substance, we extracted specific biomarker candidates for the respective mechanism of action. For atorvastatin, we identified the most highly regulated genes of the ethylene-activated signaling pathway, cellular response to lipid and glycerophospholipid metabolic process ontologies, which were not regulated by bentazon (Table S6). Similarly, for bentazon, we identified the most highly regulated genes of the ontologies cellular response to blue light, cellular response to the light stimulus, and photosynthesis, a light reaction, which were not regulated by atorvastatin (Table S6). In addition, we also selected biomarker candidates whose expression was altered by both test compounds but in different directions. Examining the expression changes of these biomarker candidates in the abbreviated*L. minor*assay developed here using rapid analyses such as RT-qPCR allows time-saving screening for the respective mechanism of action and complements the endpoints of the 7 day OECD guideline assay.

In fish, transcriptomic points of departure (tPODs) detection were recently presented as a possible approach for quantitative hazard assessment based on transcriptomics.^51^ Such an approach could in principle also be considered for other test organisms, such as*L. minor*. With the functional annotation of the*L. minor*genome, our work provides an important prerequisite for such future studies. However, for a reliable usability of tPODs for hazard assessment of substances, it still needs to be explored whether and how the relationship of tPODs to apical effects changes depending on the mechanism of action.

In summary, our study established a shortened 3 day growth inhibition assay in*L. minor*, which allows an identification of biomarker candidates for the MoA of test compounds based on gene expression signatures beyond the endpoints of the OECD guideline assay. Short-term gene expression analysis, such as RT-qPCR, of these candidate biomarkers allows this abbreviated assay to be used for screening the MoA in*L. minor*. The functional annotation of the*L. minor*reference genome developed in our study allows ORA analyses to detect functional impairment and is transferable to other poorly or unannotated organisms. The shortened assay will help develop future screening approaches for hazard assessment of compounds that can identify early MoA in*L. minor*which lead to adverse effects.

## Supporting Information Available

Additional experimental details, materials and methods including qualification schemes of RNA-Seq data, and supplemental figures and tables (PDF)
