# Supplementary material for: Short-Term Test for Toxicogenomic Analysis of Ecotoxic Modes of Action in Lemna minor
Source: Environ Sci Technol. 2022 Aug 4;56(16):11504–15. doi: 10.1021/acs.est.2c01777 (PMC9386900; doi:10.1021/acs.est.2c01777)
Supplement: Supplementary file 1 — es2c01777_si_001.pdf [file es2c01777_si_001.pdf]

A short term test for toxicogenomic analysis of ecotoxic modes of action in Lemna minor

Alexandra Loll[a,b,#], Hannes Reinwald[a,c,#], Steve U. Ayobahan[a], Bernd Göckener[d], Gabriela Salinas[e], Christoph Schäfers[f], Karsten Schlich[f], Gerd Hamscher[b], Sebastian Eilebrecht[a,]*

[a]Fraunhofer Attract Eco'n'OMICs, Fraunhofer Institute for Molecular Biology and Applied Ecology, Auf dem Aberg 1, 57392 Schmallenberg, Germany

[b]Institute of Food Chemistry and Food Biotechnology, Justus Liebig University Giessen, Heinrich-Buff-Ring 17, 35392 Giessen, Germany

[c]Department Evolutionary Ecology and Environmental Toxicology, Goethe University Frankfurt, Max-von-Laue-Straße 9, 60438 Frankfurt am Main, Germany

[d]Department of Food and Feed Safety, Fraunhofer Institute for Molecular Biology and Applied Ecology, Schmallenberg, Germany

[e]NGS-Services for Integrative Genomics, University of Göttingen, Göttingen, Germany

[f]Department Ecotoxicology, Fraunhofer Institute for Molecular Biology and Applied Ecology, Schmallenberg, Germany

[#]A.L. and H.R. contributed equally to this paper

**Email: sebastian.eilebrecht@ime.fraunhofer.de*

KEYWORDS. Transcriptomics, proteomics, biomarkers, functional annotation, HMG-CoA reductase inhibition, PSII-inhibition, *Lemna minor*

SYNOPSIS. By combining a shortened growth inhibition test with transcriptomics and proteomics, a method for rapid assessment and differentiation of modes of action in *L. minor* was developed.

1 Supplemental Materials and Methods

1.1 Chemical analysis

The concentrations of atorvastatin and bentazon in the aqueous samples were determined by chemical analysis that was performed separately for both substances by ultra-high performance liquid chromatography coupled with tandem mass spectrometry (UHPLC–MS/MS).

For analysis of atorvastatin, samples of 5 mL volume were diluted with 1 mL methanol in glass tubes. Where necessary, samples were priorly diluted with a mixture of the medium and methanol (5/1, v/v) to yield concentrations within the calibration range. Data were collected on a Waters Acquity UPLC H-Class system coupled to a Waters Xevo TQ-S tandem mass spectrometer operated in positive electrospray ionization (ESI) mode. Chromatographic separation was performed on a Waters UPLC BEH C18 column (1.7 µm, 50 mm x 2.1 mm) at a flow rate of 0.25 mL/min and a column temperature of 30 °C. The injection volume was 2 µL.

A mixture of water and methanol (95/5, v/v) with 2 mM ammonium acetate and 0.02% ammonium hydroxide was used as mobile phase (MP) A and methanol with 2 mM ammonium acetate and 0.02% ammonium hydroxide was used as MP B. The following linear gradient was applied for elution: 0 – 0.2 min: 0% MP B; 2.0 min: 20% MP B; 4.0 – 8.0 min: 100% MP B; 8.1 – 10 min: 0% MP B. The mass transition used for the quantification of atorvastatin was m/z 559.2 > m/z 440.1; the confirmation of the substance’s identity was carried out via the mass transition m/z 559.2 > m/z 250.0.

A seven-point matrix calibration in a mixture of the medium and methanol (5/1, v/v) was used in a concentration range from 0.30 µg/L to 30 µg/L (referring to the aqueous part). The coefficient of determination (r2) of the linear calibration function was determined to be >0.998. The analytical method was successfully validated for the medium on two fortification levels (1.0 and 10 µg/L) according to the EU guideline SANTE/2020/12830[1] at a limit of quantification (LOQ) of 1.0 µg/L. The accuracy (overall mean recovery) was 99.2% and the precision was 0.90% (RSD of the recovery values).

Two quality control (QC) samples with concentrations of 2.0 and 20 µg/L were used for the ongoing verification of the matrix calibration. Recoveries of QC samples were within a range of 80 – 120%. Matrix-charged procedural blanks and controls were prepared and run with the samples to exclude possible cross-contaminations during laboratory work.

Chemical analysis of bentazon was conducted with a method similar as described above. 5 mL of the aqueous sample were amended with 1 mL of methanol. If necessary, samples were further diluted with a mixture of the medium and methanol (5/1, v/v) to yield concentrations within the calibration range. A sample volume of 50 µL was directly injected into a Waters

Acquity UPLC H-Class system coupled to a Waters Xevo TQ-D system. The same chromatographic column as mentioned above was used, but with a flowrate of 0.6 mL/min and a column temperature of 35 °C. As mobile phases, water (MP A) and acetonitrile (MP B) were acidified with 0.1% formic acid each. The linear gradient program was as follows. 0 min: 5% MP B; 0.5 min: 30% MP B; 3.0 – 4.0 min: 90% MP B; 4.0 – 5.0 min: 5% MP B. The mass transition used for the quantification of bentazon was m/z 239.2 > m/z 132.1. The mass transition m/z 239.2 > m/z 174.9 was used for confirmatory purposes.

A six-point matrix calibration with a concentration range from 1.0 µg/L to 10 µg/L (referring to the aqueous part) was prepared with the same solvent ratios as the samples. The coefficient of determination (r2) of the quadratic calibration function was determined to be >0.997.

1.2 RNA extraction

RNA extraction was performed according to *RapidPURE RNA Plant Kit* (REF 112722050 – MP Biomedicals Illkirch, France), with exceptions explained in the following. Since less plant material (25 mg) was used for extraction, the added amounts of Lysis Solution PS and ethanol were halved so that all lysate could subsequently be transferred to the spin filter in one step. The homogenization step after adding the Lysis Solution PS was performed at 5 m/s for one minute. Beyond that, the number of washing steps was optimized to three steps with Wash Buffer No. 1 and two steps of washing with Buffer No. 2. Subsequently, the samples were centrifuged at maximum speed for 2 min to eliminate any traces of ethanol.

1.3 Transcriptomics

Sequencing libraries were prepared for each sample (each normalized to 100 ng/µl total RNA) at the sequencing facility "NGS-Services for Integrative Genomics" at the University of Göttingen in Germany. According to their standard workflow, Poly(A)+ RNA was purified, fragmented, and transcribed into cDNA for library preparation using the *TruSeqRNA Library Prep Kit (v2)* (Illumina, UK) following the manufacturer's instructions. Libraries were validated using a Fragment Analyzer system (Agilent, Santa Clara, USA) before sequencing. Sample libraries were sequenced on an Illumina HiSeq 4000 system in 50 bp single read mode with approximately 30 million raw reads per sample.

Sequence images were transformed to BCL files with Illumina BaseCaller software and demultiplexed to fastq files via bcl2fastq (v2.17.1.14). Adapter sequences were removed using trimmomatic (v0.39) and sequencing quality of each sample was assessed using FastQC (v0.11.5). Additionally, reads were checked for potential contaminations by FastQ Screen (v0.14.1) using bowtie2 (v2.3.2) against the following organism's reference genomes: *Oryzia latipes, Danio rerio, Oncorhynchus mykiss, Daphnia magna, Homo sapiens, Mus musculus,*

Drosophila melanogaster, Saccharomyces cerevisiae, Eschericia coli. For those organisms possible, pre-built Bowtie2 indices were directly downloaded from Babraham Bioinformatics with the built in function ‘fastq_screen --get_genomes’, which also included custom build human rRNA database.

Sequence reads were aligned to the *Lemna minor* reference genome 2019v2 (www.lemna.org) via STAR allowing for 2 mismatches within 50 bases. A respective genome annotation files in GFF format was obtained from the CoGe database (www.genomevolution.org) under: https://genomevolution.org/coge/LoadGenome.pl?wid=47218 (requires CyVerse account for access). For better downstream analysis compatibility, the GFF annotation file was converted to GTF using the `agat_convert_sp_gff2gtf.pl` function from the AGAT toolkit [https://github.com/NBISweden/AGAT]. Contig headers in the reference genome fasta were harmonized to the respective annotation file. Final versions of compatible reference genome fasta and matching gtf annotation file used in this study are publicly accessible on Zenodo under the accession 6045874 [https://zenodo.org/record/6045874]. Alignment quality was evaluated via RSeQC, Qualimap and Samtools. Feature mapped reads were counted through featureCounts. A collective quality report summary on sequence reads, alignment and mapped features was generated using MultiQC. Subsequently, gene count library normalization and differential gene expression analysis (DGEA) was conducted as described in the main manuscript.

1.4 Protein extraction and peptide labelling

Protein extraction was fulfilled simultaneously to RNA extraction according to the manufacturer’s protocol (*RapidPURE RNA Plant Kit*). In conclusion of the optimized volumina used for RNA extraction, less of the protein containing flow through was contained. Therefore, the solution was split into two equal amounts of about 280 µL and a threefold volume of ice-cold acetone as well as 350 µL of ice-cold ethanol (50%) were added for precipitation. Addition of acetone was followed by 10 min, addition of ethanol by 3 min of centrifugation at 13,400 g and 4°C.

The protein pellet was stored at -20°C until further processing. To this, 900 µL of a lysis buffer (6 M urea, 2 M thiourea, 4% CHAPS in 50 mM TEAB; pH = 8) were added and then incubated for 1 h at 4°C for resolubilization. Samples were then sonicated and centrifuged at 14,000 g for 15 min. In order to change the buffer, 450 µL were transferred to a MWCO filter (Amicon Ultra 3K, 0,5 mL, Merck). To this end, 450 µL of Cleanup Buffer (2 M urea, 0,2% SDS; pH = 8,4) and 100 mM TEAB were added sequentially. According to the manufacturer’s recommendation, each concentration step was completed by centrifugation at 14,000 g for 30 min. Protein washing by addition of Cleanup Buffer was performed four times, and 100 mM TEAB was added twice. After that, the remaining sample volume of approximately

35 µL was briefly centrifuged at 1,000 g for one minute into a new tube. Protein quantification followed the manufacturer's instructions using the *Pierce BCA Protein Assay Kit* (REF 23225 – Thermo Scientific, USA). The subsequent workflow for labelling 25 µg of tryptic digested protein samples (1:40; trypsin : protein) with a *TMT-6plex* (Thermo Scientific, USA) was performed as recommended by the manufacturer. The workflow was modified after the protocol described in Ayobahan et al. 2019. Reduction, alkylation and acetone precipitation (overnight) was performed with 100 µL of protein samples normalized to a concentration of 1 µg/µL. Pellets were washed with 500 µL 70% ethanol and dried for 3 minutes before they were resuspended in 100 µL 50 mM TEAB with sonication for 20 s at 35 kHz and digested with 2.5 µg (2.5 µL) trypsin overnight at 37°C. Digested peptides were quantified using *BCA peptide quantification assay* (Thermo Scientific, USA) following the manufacturer's instructions. 25 µg of digested peptides were then subjected to TMT labelling (*TMT 6-plex kit*) with the respective amount of labels (13.6 µL) as recommend by the manufacturer. The distribution of isobaric labels across the replicates per tested substance is given below:

	TMT-126 or TMT-129	TMT-127 or TMT-130	TMT-128 or TMT-131
Replicate 1	NC	LE	HE
Replicate 2	HE	NC	LE
Replicate 3	LE	HE	NC

After labelling, samples were combined and dried in a SpeedVac drier before being resuspended in 100 µL sample buffer (0.5% FA , 5% ACN in ultra-pure water) via sonication for 60 s at 35 kHz. Combined samples were cleaned from excessive TMT labels via C18 cleanup protocol as described by the manufacturer (Thermo Scientific, USA). After C18 cleanup, excessive ACN was removed by drying samples in a SpeedVac. Lastly, the dry pooled label peptide sample was dissolved in LC-MS buffer (2% ACN, 0.1% FA) by sonication for 60 s at 35 kHz. Peptide concentrations were measured via *BCA peptide quantification assay.* For LC-MS/MS measurement, pooled label peptide sample was adjusted to 500 ng/µL.

1.5 Proteomics

1.5.1 nanoLC-MS/MS analysis

The tryptic peptides were resuspended in 0.1% formic acid (solvent A) and analyzed on a Thermo Fisher Q Exactive mass spectrometer (MS) (Thermo Fisher, Waltham, USA) as described previously in Ayobahan et al[2,3]. The MS was equipped with a nanoflow ionization source and coupled to a nanoACQUITY UPLC (Waters, Massachusetts, USA). Data dependent acquisition was performed in a positive ion mode with the electrospray voltage set

at 1.8 kV. For quantitative measurement, 500 ng of each replicate mixtures were injected onto nanoACQUITY UPLC packed 20 mm x 180 µm diameter C18 Trap Column, heated at 40 °C. Upon trapping, the peptides were eluted onto a nanoACQUITY reversed-phase analytical column (25-cm length, 75-µm i.d.) (Waters, Massachusetts, USA) using a linear gradient from 3-97% (v/v) of 90% (v/v) acetonitrile in 0.1% (v/v) formic acid (solvent B) for 170 minutes with a flow rate of 300 nL/min. The full MS survey scans were acquired at 375-1500 m/z range, using a resolving power of 70,000 at 200 m/z for the MS and 35,000 for MS2 scans. Fragmentation was triggered for the top 10 precursors of charge state ranging from 2+ to 7+ and intensity threshold above 2E4. Dynamic exclusion was set to exclude previous sequenced precursor ions for 30 seconds within a 10-ppm window. The automatic gain control and maximum injection time for MS2 spectra were set at 1E5 and 200 ms, respectively. MS2 scans were acquired in centroid mode. MS calibration was performed using the LTQ Velos ESI Positive Ion Calibration Solution (ThermoFisher, Waltham, USA).

1.5.2 Bioinformatics of proteome data

The resulting MS/MS data were processed using Maxquant search engine (v.2.0.1.0), at a peptide-spectrum match FDR of < 1 %. Tandem mass spectra were matched to a custom protein database with predicted protein sequence from the *L. minor* reference genome combined with duckweed related protein sequences (including pro- and eukaryotic organism) obtained from Uniprot (search term “duckweed”). That way, protein sequences of bacteria with known duckweed association were also included in the PSM search database. Further, a common lab contaminant protein list was provided for the PSM search. Precursor mass tolerance of ±20 ppm and the integration of fully tryptic peptides with up to two missed cleavage sites were applied in the database search. Cysteine carbamidomethyl, peptide N-terminus TMT6plex and lysine TMT6plex were set as static modifications, whereas the acetylation of protein N-terminal and the oxidation of methionine were included as variable modifications. Only unique and razor peptides with no importation of missing values were considered. Differentially expressed proteins were identified using the MSstatsTMT R package version 2.2.0 on the basis of three technical replicate measurements of three biological replicates per condition[4]. The measured intensities of the isotope-labelled peptides were first log2-transformed. Thereafter, a reference channel-based normalization was applied by computing an average signal sum as the pseudo-reference channel, to remove any potential technical variation across runs. Proteins were tested for significant differences in expression to the non-treated control group using MSstatsTMT’s implemented linear-mixed model with a moderated t-statistic. Proteins were considered statistically significantly regulated for BH-corrected p-values (padj) < 0.05[5] with degrees of freedom (DF) ≥ 6. The mass spectrometry proteomics

data have been deposited in the ProteomeXchange Consortium via the PRIDE partner repository[6] with the dataset identifiers PXD031680 (atorvastatin) and PXD031679 (bentazon).

1.6 Functional *L. minor* genome annotation and overrepresentation analysis (ORA)

A complementary approach using BLAST[32] and eggNOG[33] was applied to annotate genes of the *L. minor* reference genome (2019v2) with gene ontology (GO) terms and gene descriptors based on protein sequence homology. A general workflow overview is shown in the main manuscript Figure 5. First, a multi fasta file listing all theoretical coding sequence (CDS) per gene was extracted from the reference genome with the corresponding GTF file [Zenodo 6045874] using cufflink's *gffread* function, which also supports GTF format. The CDS were then translated into their respective amino acid sequences using *transeq* from the EMBOSS package.

For the BLAST-based annotation, first a local search database was created. Therefore, the reference proteomes of well annotated reference plant species (*Arabidopsis thaliana, Sorghum bicolor, Phaleanopsis equestris, Oryza sativa, Triticum dicoccum, Zea mays*) as well as all available protein sequence entries for duckweed species (e.g. *Spirodela* sp., *Landolita* sp., *Wolffia* sp., *Lemna* sp.) and duckweed-associated prokaryotes were downloaded from from the Uniprot database.[34]. Translated *L. minor* CDS sequences were then subjected to a *blastp* search against this database and each gene was matched with the Uniprot ID of the best hit scored by %-alignment for each reference plant species. The subsequent steps were performed in R. Results were cleaned from non-plant related top hits as well as alignment lengths < 20 amino acids and alignment similarities < 35%. Each *L. minor* gene ID from these cleaned results was then annotated with the combined set of unique GO terms associated with the matched Uniprot IDs across the plant taxa. GO terms for the filtered Uniprot ID blastp hits were obtained from Uniprot. For the eggNOG-based annotation, translated *L. minor* sequences were subjected to the eggNOG annotation pipeline with default settings.[35] All non-plant related matches were removed from the eggNOG annotation, before results were merged with filtered blastp search results. Hereby, GO terms from both searches were combined and the resulting gene2GO table (one gene mapped to multiple GO terms) was converted to a GO2gene format (one GO term mapped to multiple genes) using the *topGO* package function *inverseList()*. GO2gene table was filtered from deprecated GO terms while annotating the GO ids with GO term descriptions and ontology features (BP, MF or CC) using the *clusterProfiler* package[36] functions *go2term()* and *go2ont().* To allow for easy implementation of the GO term annotation for *L. minor* in the powerful enrichment analysis tool clusterProfiler, an custom AnnotationDbi organism package was built using the AnnotationForge package function *makeOrgPackage()*. The *org.Lminor.eg.db* package is publicly available under Zenodo accession 6045874 (www.zenodo.org).

Overrepresentation analysis (ORA) was conducted in R using clusterProfiler v3.18[36] package via the *enrichGO()* function. ORA for significantly enriched biological process (BP) GO terms was performed for each of the tested substances with their core DEG set (genes both identified as differentially expressed in high and low exposure condition). The total universe background of 15278 genes were the common set of Lemna gene IDs among all count libraries after low gene counts removal. The *orgDb* parameter was specified as *"org.Lminor.eg.db"* with *keyType="GID".* *p* value adjustment method for multiple testing was performed after BH. Terms with *p.adjust* ≤ 0.05 were considered significantly enriched. To prepare GO terms for measuring semantic similarities among them, the *godata()* function from the GOSemSim package was used with the above specified *OrgDb* and *keytype.* To build significantly enriched network plots via *emapplot()* for data exploration purposes, semantic similarities between GO terms were computed after *"Wang".*

2 Supplemental tables

Table S1. pH measurements of the pre-tests of atorvastatin and bentazon on test start and end.

pH	atorvastatin		bentazon	
	day 0	day 7	day 0	day 7
Control	5.67	6.55±0.07	5.57	6.63±0.06
c_1 (ATV: 0.001 mg/L; BTZ: 1.0 mg/L)	5.69	6.26±0.07	5.58	6.33±0.17
c_2 (ATV: 0.01 mg/L; BTZ: 1.5 mg/L)	5.74	6.36±0.20	5.58	6.28±0.21
c_3 (ATV: 0.1 mg/L; BTZ: 2.0 mg/L)	5.74	6.51±0.02	5.57	6.26±0.14
c_4 (ATV: 1.0 mg/L; BTZ: 3.0 mg/L)	5.75	6.11±0.03	5.57	6.14±0.25

Table S2. Light intensity, temperature and pH conditions of the start and end of the modified *Lemna sp.* Growth inhibition test with atorvastatin and bentazon.

parameter	atorvastatin		bentazon	
	day 0	day 3	day 0	day 3
light intenisty [µmol·m^{-2}·s^{-1}]	127.1	136.3	129.6	140.1
temperature [°C]	23.9	24.6	24.4	23.6
pH (Control)	5.70	5.83±0.05	5.74	6.03±0.15
pH (EC_5)	5.65	5.81±0.02	5.67	5.85±0.06
pH (EC_{20})	5.59	5.77±0.02	5.09	5.42±0.09

Table S3. Obtained effect concentrations as mass concentration and molarity of each substance. [lower 95%-cI – higher 95%-cI]

substance	EC_5	EC_{10}	EC_{20}	EC_{50}
Atorvastatin	0.03 mg/L [0.001 – 0.082]<br>0.027 µM<br>[0.001 – 0.074]	0.05 mg/L [0.003 – 0.116]<br>0.045 µM<br>[0.003 – 0.104]	0.09 mg/L [0.013 – 0.184]<br>0.081 µM<br>[0.012 – 0.165]	0.27 mg/L [0.119 – 0.692]<br>0.242 µM<br>[0.107 – 0.621]
Bentazon	0.69 mg/L [0.678 – 0.695]<br>2.875 µM<br>[2.825 – 2.896]	0.83 mg/L [0.819 – 0.837]<br>3.458 µM<br>[3.413 – 3.488]	1.04 mg/L [1.025 – 1.051]<br>4.333 µM<br>[4.271 – 4.379]	1.60 mg/L [1.577 – 1.624]<br>6.667 µM<br>[6.571 – 6.767]

Table S4. Obtained effect concentrations as mass concentration and molarity for the frond number of *L. minor* after treatment with atorvastatin and bentazon. [lower 95%-cI – higher 95%-cI]

substance	EC_5	EC_{10}	EC_{20}	EC_{50}
ATV	0.02 mg/L<br>[0.000 – 0.067]<br>0.018 µM<br>[0.000 – 0.060]	0.03 mg/L<br>[0.000 – 0.104]<br>0.027 µM<br>[0.000 – 0.093]	0.08 mg/L<br>[0.003 – 0.186]<br>0.072 µM<br>[0.003 – 0.167]	0.35 mg/L<br>[0.120 – 1.161]<br>0.314 µM<br>[0.108 – 1.041]
BTZ	1.11 mg/L<br>[0.663 – 1.375]<br>4.620 µM<br>[2.759 – 5.722]	1.29 mg/L<br>[0.859 – 1.536]<br>5.369 µM<br>[3.575 – 6.393]	1.54 mg/L<br>[1.169 – 1.768]<br>6.409 µM<br>[4.865 – 7.358]	2.19 mg/L<br>[1.938 – 2.519]<br>9.114 µM<br>[8.066 – 10.484]

Table S5. Integration of transcriptome and proteome data. The numbers of detected and differentially expressed genes (DEG) in each condition are indicated per test compound and omics methodology. In the case of transcriptomics, the DEG numbers do not include an effect size (lfc) cut-off, but only a significance threshold of $p_{adj} \leq 0.05$. For comparability of DEG sets at the proteome and the transcriptome level, quadrant count ratios (QCR) are given for the overlap, where possible and reasonable.

	**atorvastatin**				**bentazon**			
	**transcr.**	**prot.**	**overlap**	**QCR**	**transcr.**	**prot.**	**overlap**	**QCR**
**detected**	15278	1178	984	NA	15278	1178	984	NA
**DEG EC_5**	152	204	2	1.00	3860	681	240	0.37
**DEG EC_{20}**	1308	252	10	0.40	6649	893	509	0.64
**DEG EC_5 & EC_{20}**	123	91	0	NA	3219	585	177	0.63

Table S6. Biomarker candidates for the modes of action of both test substances. The gene ID, the annotation of the best match of our functional annotation pipeline, the log_2-fold change values at the transcriptome level after exposure to the EC20 of each test compound as well as the affiliation to the MoA-associated gene ontologies are indicated.

	ID	annotation	log2-fold change Atorvastatin	log2-fold change Bentazon	ethylene-activated signaling pathway	cellular response to lipid	glycerophospholipid metabolic process	cellular response to blue light	cellular response to light stimulus	photosynthesis, light reaction
**Atorvastatin**	Lminor_014132	Copper-transporting ATPase RAN1	-1,25	0,01	■					
	Lminor_018118	Putative leucine-rich repeat receptor-like protein kinase family protein	-0,43	0,05		■				
	Lminor_013682	Hydrolase family protein / HAD-superfamily protein	-0,87	0,17			■			
**both**	Lminor_003253	BTB and TAZ domain protein 2	-1,34	1,91		■				
	Lminor_008256	Regulatory protein opaque-2	-1,34	1,75		■				
	Lminor_018262	Glycerophosphodiester phosphodiesterase GDPD2	-0,74	1,88			■			
	Lminor_013527	Trehalose 6-phosphate phosphatase	-1,27	1,16						
	Lminor_004696	DUF3700 domain-containing protein	-1,68	3,69						
**Bentazon**	Lminor_014291	J domain-containing protein required for chloroplast accumulation response 1	0,04	2,00				■	■	
	Lminor_015609	Early light inducible protein1	-0,12	-1,63				■	■	
	Lminor_004499	Early flowering 3-B1	0,09	1,43					■	
	Lminor_005647	Cysteine-rich repeat secretory protein 3	-0,23	-2,26						■
	Lminor_012386	Protein HIGH CHLOROPHYLL FLUORESCENCE PHENOTYPE 173, chloroplastic	-0,09	1,46						■
	Lminor_008448	Protease Do-like 1 chloroplastic	0,01	1,09						■

3 Supplemental figures

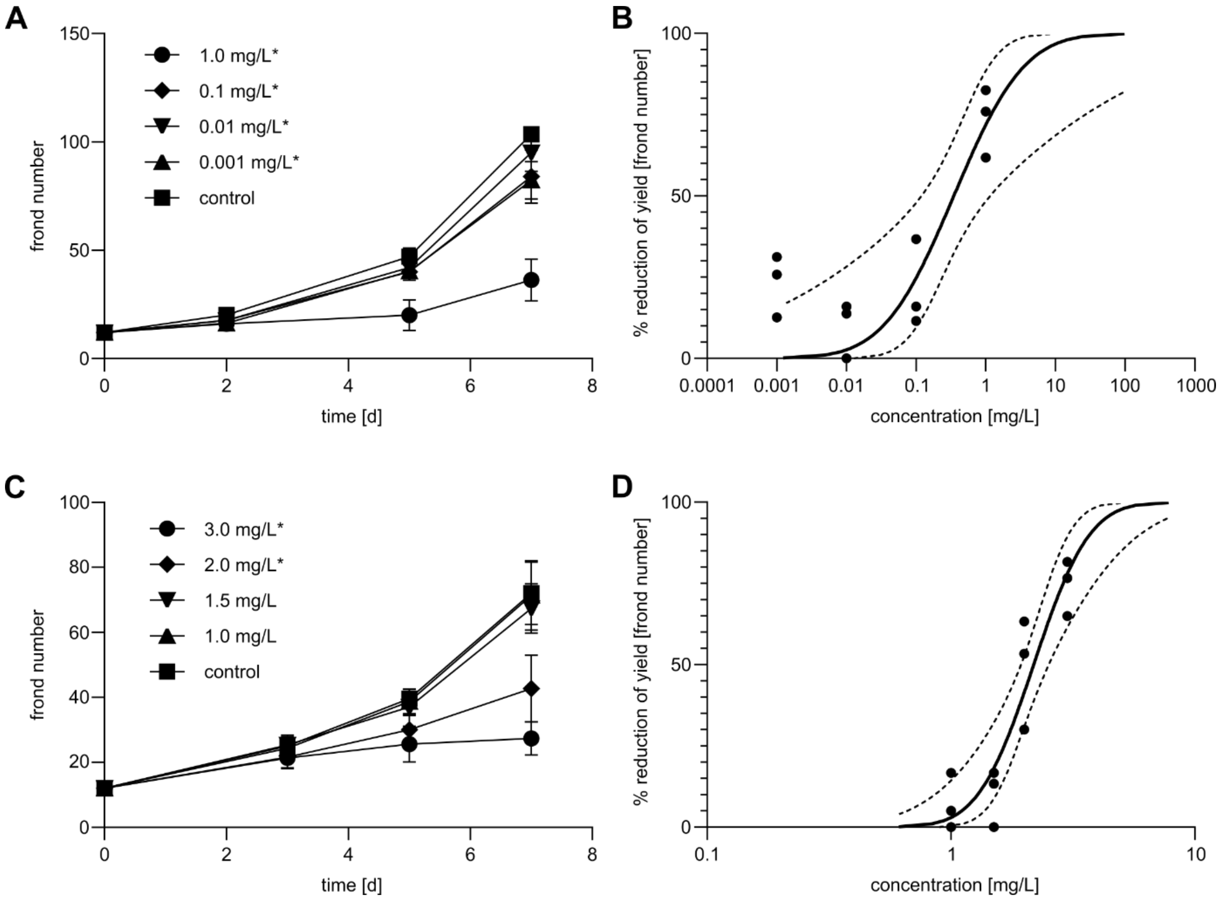

Figure S1. Pretest for detecting low effect concentrations of atorvastatin and bentazon according to OECD TG 221. **(A)** Time-dependent course of the frond number at different exposure concentrations of atorvastatin. Statistically significant changes compared to the control are indicated by an asterisk (Williams Multiple Sequential t-test). The standard deviation is given as error bar. **(B)** Concentration-response curve of frond number yield reduction after exposure to atorvastatin on day 7. **(C)** Time-dependent course of the frond number by different exposure concentrations to bentazon. Statistically significant changes compared to the control are indicated by an asterisk (Williams Multiple Sequential t-test). The standard deviation is given as error bar. **(D)** Concentration-response curve of frond number yield reduction after exposure to bentazon on day 7.

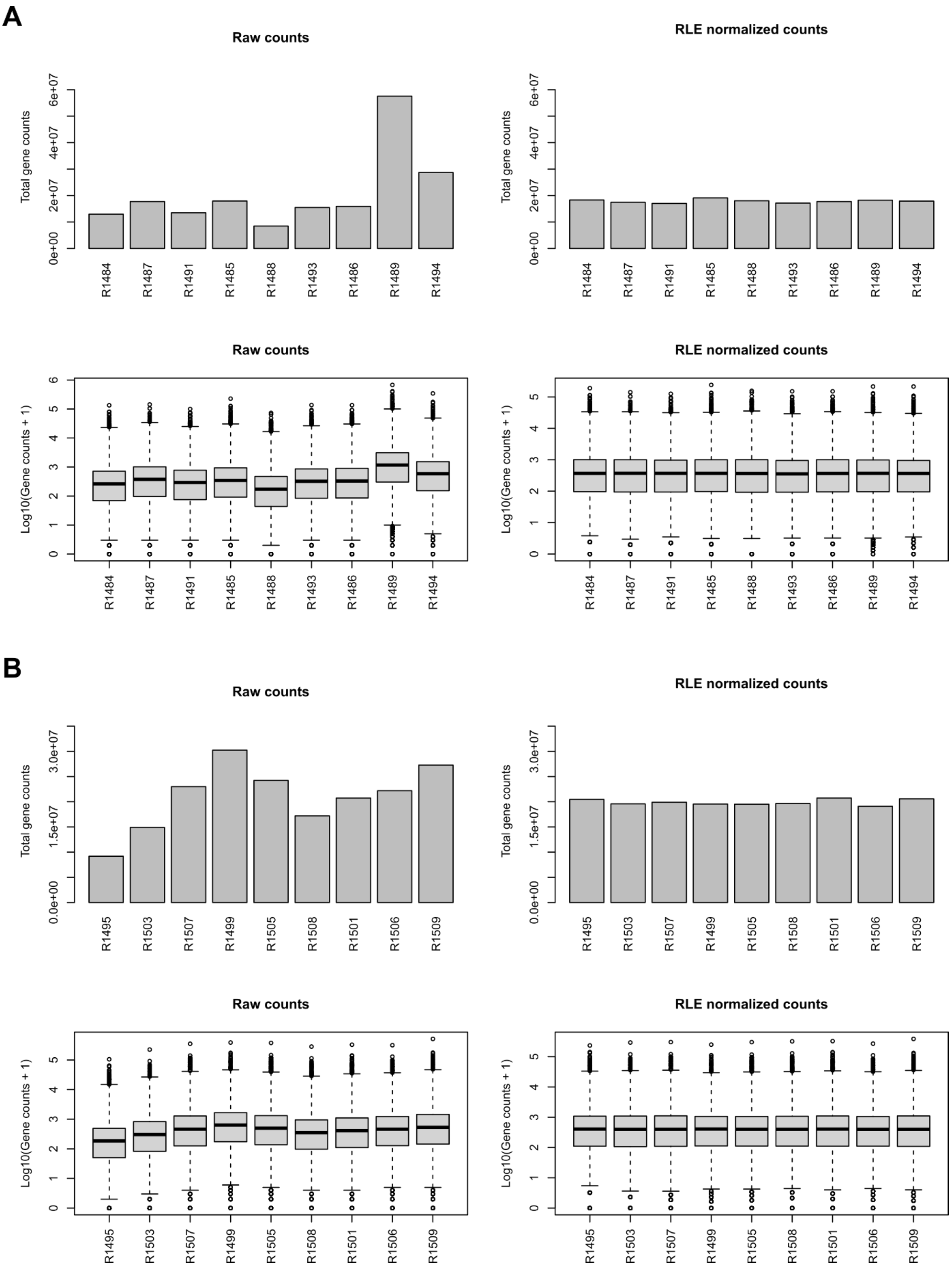

Figure S2. RNA-Seq read count normalization using DESeq2. **(A)** Raw and relative log expression (RLE) normalized read counts of atorvastatin treated samples (EC_5 and EC_{20}) and control samples. **(B)** Raw and relative log expression (RLE) normalized read counts of bentazon treated samples (EC_5 and EC_{20}) and control samples.

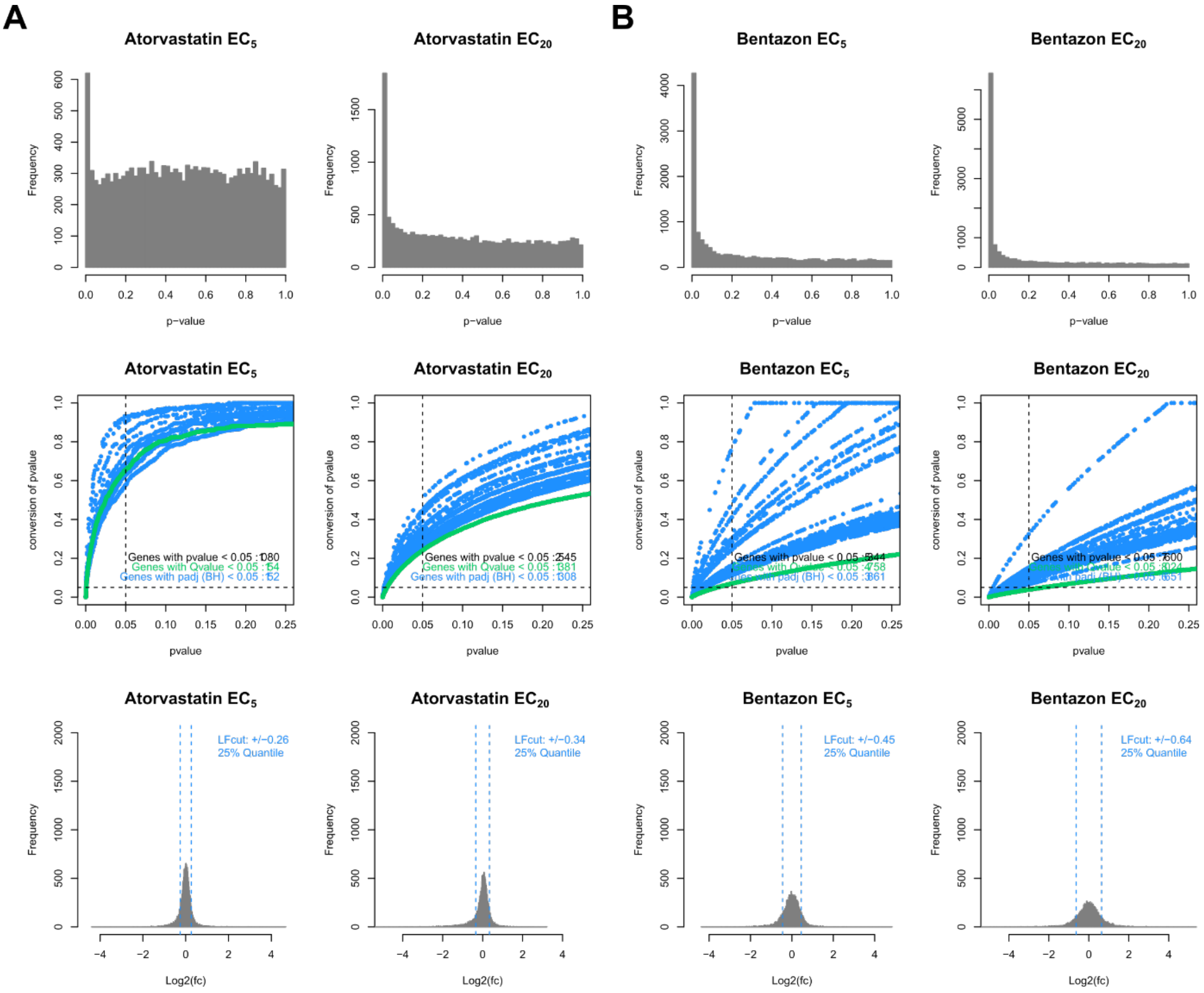

Figure S3. Distributions of p-values, p-value conversion and lfc distributions after exposure to atorvastatin and bentazon compared to the control as observed by gene expression data. **(A)** Top: Distribution of p-values of all genes after exposure to EC_5 (left) and EC_{20} (right) of atorvastatin. Centre: Conversion of p-values for all genes after exposure to EC_5 (left) and EC_{20} (right) of atorvastatin. Bottom: Distribution of lfc values of all genes after exposure to EC_5 (left) and EC_{20} (right) of atorvastatin. The lfc cut-off is indicated as dotted line. **(B)** Top: Distribution of p-values of all genes after exposure to EC_5 (left) and EC_{20} (right) of bentazon. Centre: Conversion of p-values for all genes after exposure to EC_5 (left) and EC_{20} (right) of bentazon. Bottom: Distribution of lfc values of all genes after exposure to EC_5 (left) and EC_{20} (right) of bentazon. The lfc cut-off is indicated as dotted line.

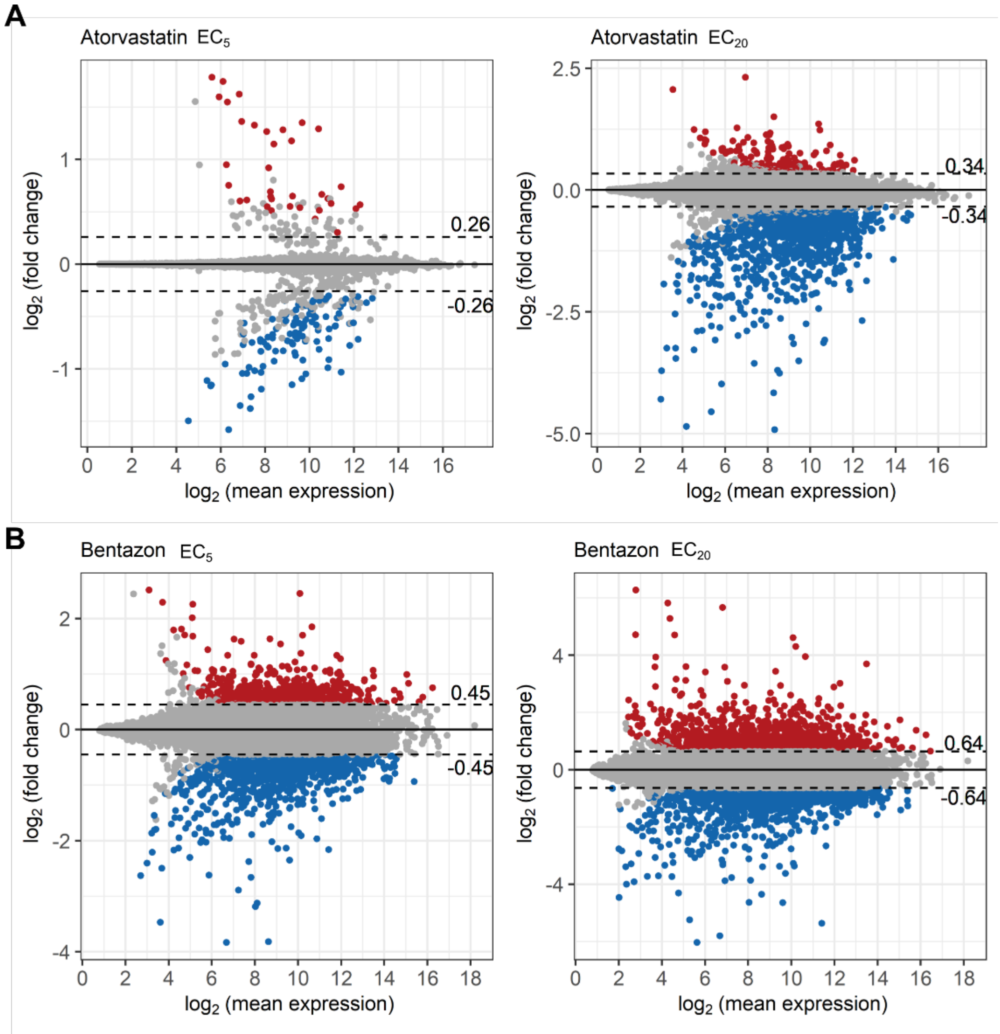

Figure S4. MA-plot illustration of the apeglm-shrunk lfc values for the genes of both conditions (EC_5 and EC_{20}) of atorvastatin **(A)** and bentazon **(B)** treatments.

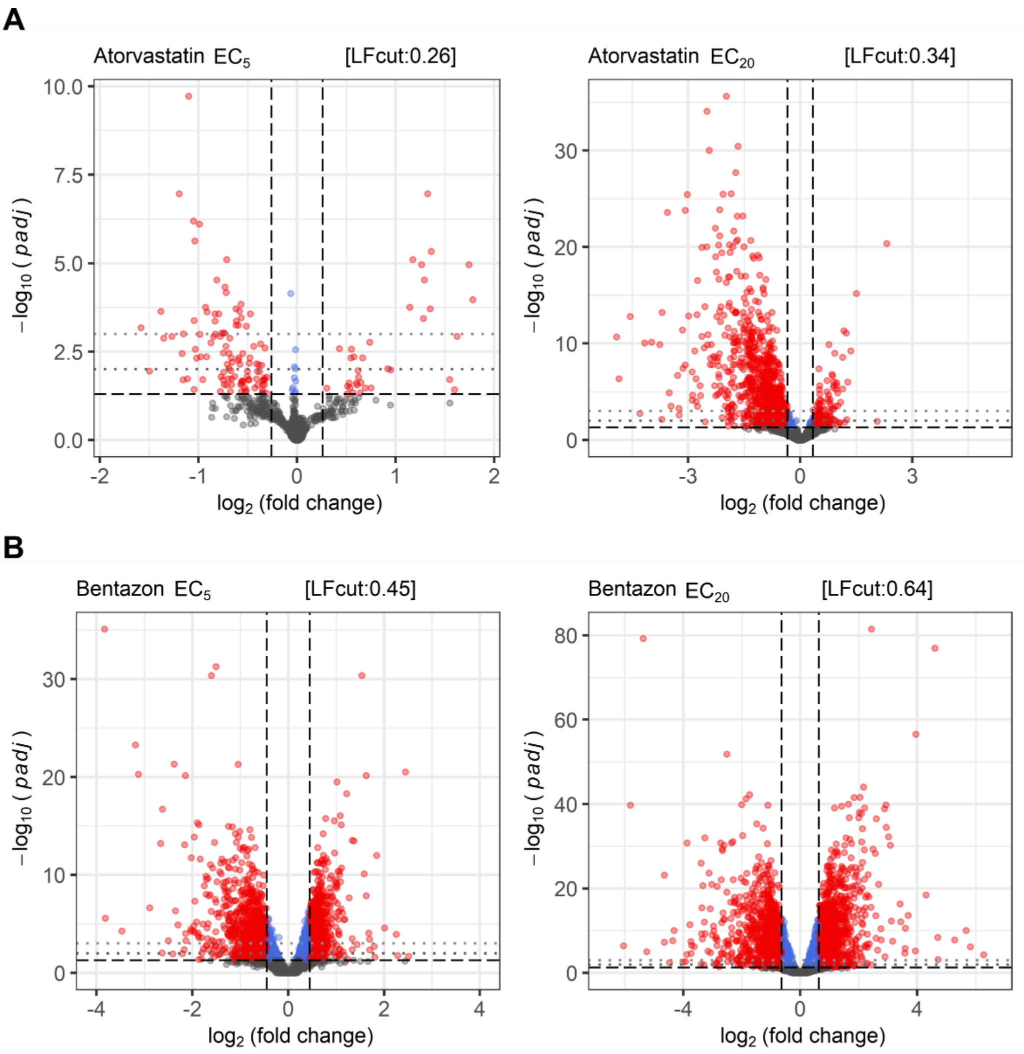

Figure S5. Vulcano plot illustration of the lfc values against the corresponding $–\log_{10}(p_{adj})$ values of genes that were differentially expressed after exposure of the EC_5 (left) and EC_{20} (right) of atorvastatin **(A)** and bentazon **(B)**. The lfc value cut-off as well as the p_{adj} cut-off are indicated as dotted lines. Genes applying to both of them are coloured red.

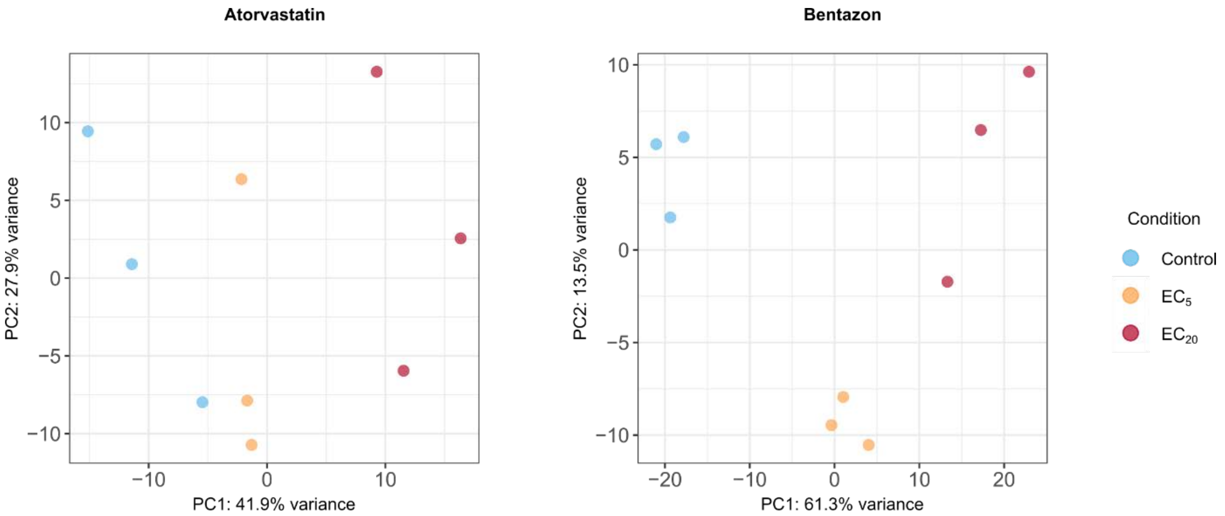

Figure S6. Principle component analysis (PCA) of control replicates and samples after treatment with EC_5 and EC_{20} of atorvastatin (left) and bentazon (right). Biological replicates are indicated as colour code.

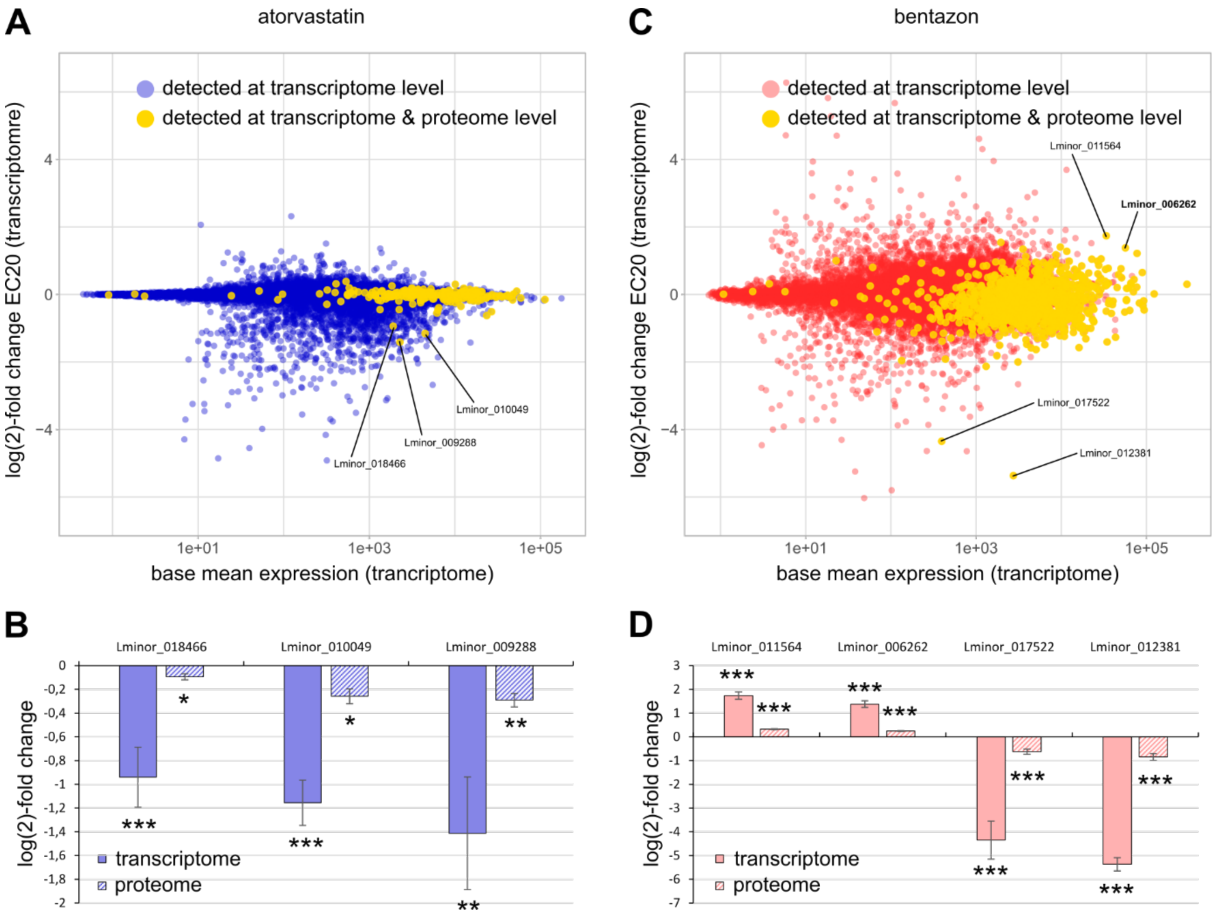

Figure S7. Transcriptome and proteome integration. **(A)** MA-plot illustration of the apeglm-shrunk lfc values for the genes of the EC_{20} of atorvastatin at the transcriptome level. Genes, which were also detected at the protein level are highlighted in yellow. For such genes detected at the proteome and the transcriptome level with relatively high log(2)-fold change values, gene identifiers are given. **(B)** Log(2)-fold change values of the genes indicated in (A) at the transcriptome and the proteome level after exposure to the EC_{20} of atorvastatin. The standard error and significance is indicated. *$p_{adj} \leq 0.05$; **$p_{adj} \leq 0.01$; ***$p_{adj} \leq 0.001$ **(C)** as in (A), but for bentazon. **(D)** Log(2)-fold change values of the genes indicated in (B) at the transcriptome and the proteome level after exposure to the EC_{20} of bentazon. The standard error and significance is indicated. *$p_{adj} \leq 0.05$; **$p_{adj} \leq 0.01$; ***$p_{adj} \leq 0.001$

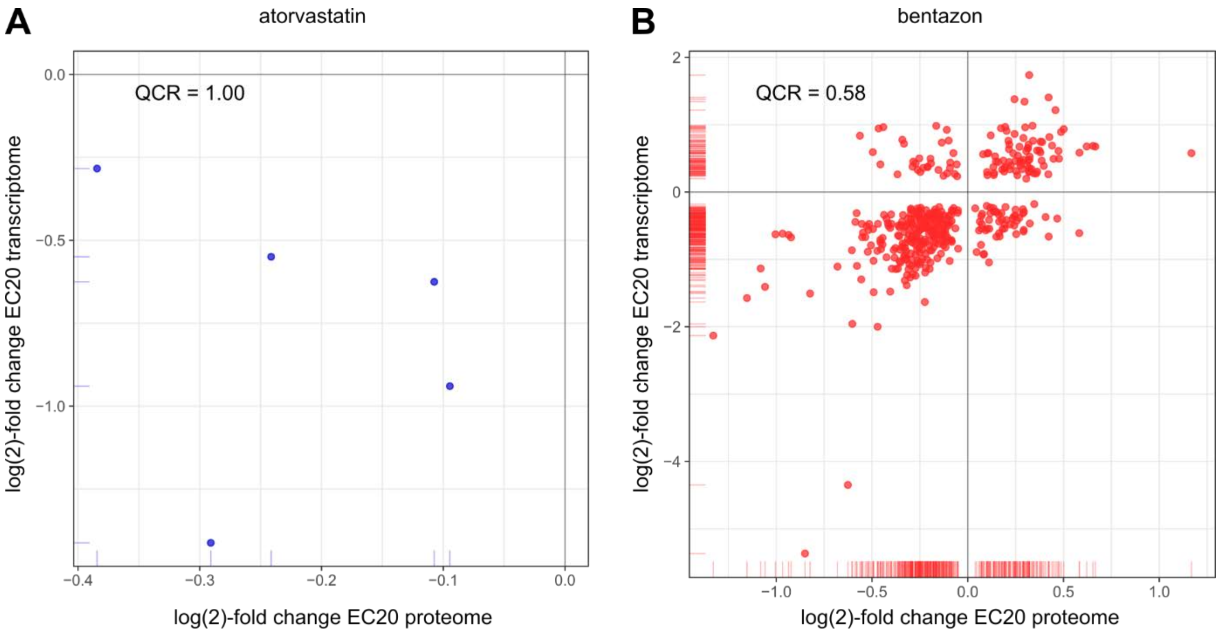

Figure S8. Scatter plot comparisons of the common sets of EC5 and EC20 target genes at the proteome level with their expression changes at the transcriptome level. **(A)** Scatter plot comparing the expression change of atorvastatin-responsive genes at the proteome level with their expression change at the transcriptome level. The quadrant count ratio (QCR) is given as a measure of similarity. **(B)** as in (A), but for bentazon.

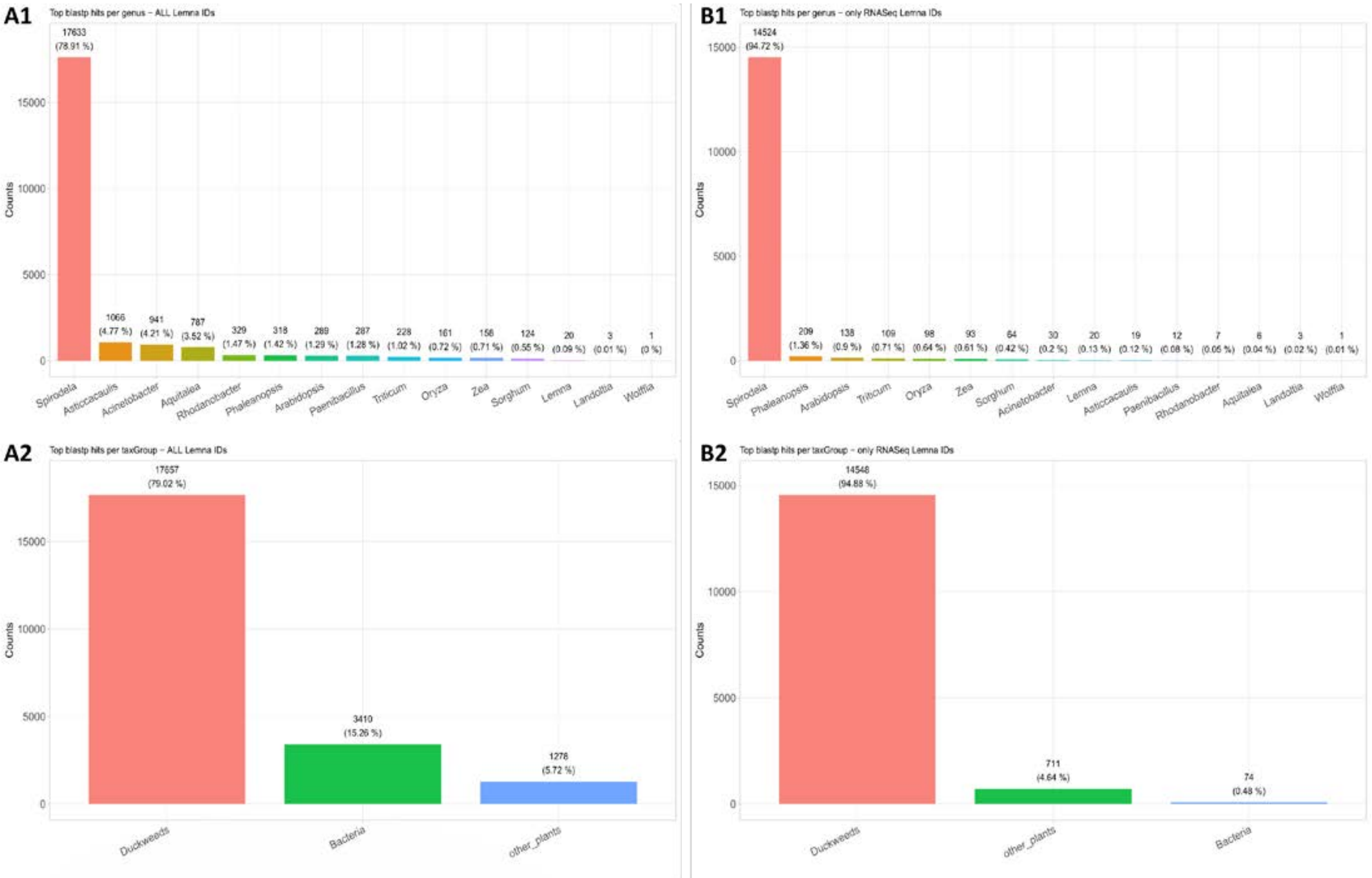

Figure S9. Total *blastp* top hit search results (**1**) per genus and (**2**) general taxa groups of “duckweeds”, “other plants” and “bacteria”. (**A)** panels show the top search result hits distribution across all 22345 *Lemna minor* gene IDs from the reference genome. (**B)** panels show the distribution only for 15333 *Lemna minor* gene IDs present in the RNA-Seq gene count libraries after removal of zero gene counts. Hence, **B2** indicates how many of the gene IDs observed in our transcriptomic dataset match with a respective taxa group. We see that in the mRNA Seq data, 99.52% of gene IDs match with duckweed related taxa (94.88%) or other plants (4.64%). Only 0.48% in this set have a top hit with a bacteria related protein sequence. In contrast, in **B1** of all matching IDs from the reference genome, a total of 15.26% has a top hit with bacteria related proteins. This suggests that the *Leman minor* reference genome still contains a relatively large fraction of contaminating bacterial sequences. This might derive from the fact that *Lemna* sp. lives in close relationship with prokaryotes such as *Asticcacaulis* sp., *Acinetobacter* sp., *Aquitalea* sp. or *Rhodanobacter* sp. The largest portion of top hits can be seen for *Spirodela* sp*.* This is not surprising, as *Spirodela intermedia* has by far the largest number of entries among all duckweeds in the Uniprot database. Unfortunately, the vast majority of these entries is not functionally annotated to any GO terms. Therefore we need to relay on functional annotations from 2nd best hits in a better studied reference plant species.

4 References

(1) European Commission. Guidance Document on Pesticide Analytical Methods for Risk Assessment and Post-Approval Control and Monitoring Purposes. *Sante/2020/12830* **2021**, *Rev. 1*.

(2) Ayobahan, S. U.; Eilebrecht, E.; Kotthoff, M.; Baumann, L.; Eilebrecht, S.; Teigeler, M.; Hollert, H.; Kalkhof, S.; Schäfers, C. A Combined FSTRA-Shotgun Proteomics Approach to Identify Molecular Changes in Zebrafish upon Chemical Exposure. *Sci. Rep.* **2019**, *9* (1), 1–12. https://doi.org/10.1038/s41598-019-43089-7.

(3) Ayobahan, S. U.; Eilebrecht, S.; Baumann, L.; Teigeler, M.; Hollert, H.; Kalkhof, S.; Eilebrecht, E.; Schäfers, C. Detection of Biomarkers to Differentiate Endocrine Disruption from Hepatotoxicity in Zebrafish (Danio Rerio) Using Proteomics. *Chemosphere* **2020**, *240*, 1–12. https://doi.org/10.1016/j.chemosphere.2019.124970.

(4) Huang, T.; Choi, M.; Tzouros, M.; Golling, S.; Pandya, N. J.; Banfai, B.; Dunkley, T.; Vitek, O. MSstatsTMT: Statistical Detection of Differentially Abundant Proteins in Experiments with Isobaric Labeling and Multiple Mixtures. *Mol. Cell. Proteomics* **2020**, *19* (10), 1706–1723. https://doi.org/10.1074/mcp.RA120.002105.

(5) Diz, A. P.; Carvajal-Rodríguez, A.; Skibinski, D. O. F. Multiple Hypothesis Testing in Proteomics: A Strategy for Experimental Work. *Mol. Cell. Proteomics* **2011**, *10* (3), 1–10. https://doi.org/10.1074/mcp.M110.004374.

(6) Perez-Riverol, Y.; Csordas, A.; Bai, J.; Bernal-Llinares, M.; Hewapathirana, S.; Kundu, D. J.; Inuganti, A.; Griss, J.; Mayer, G.; Eisenacher, M.; Pérez, E.; Uszkoreit, J.; Pfeuffer, J.; Sachsenberg, T.; Yilmaz, Ş.; Tiwary, S.; Cox, J.; Audain, E.; Walzer, M.; Jarnuczak, A. F.; Ternent, T.; Brazma, A.; Vizcaíno, J. A. The PRIDE Database and Related Tools and Resources in 2019: Improving Support for Quantification Data. *Nucleic Acids Res.* **2019**, *47* (D1), D442–D450. https://doi.org/10.1093/nar/gky1106.
